# Neoadjuvant chemo-immunotherapy is improved with a novel pulsed electric field technology in an immune-cold murine model

**DOI:** 10.1371/journal.pone.0299499

**Published:** 2024-03-25

**Authors:** Chiara Pastori, Ebtesam H. O. Nafie, Mukta S. Wagh, Stephen J. Hunt, Robert E. Neal

**Affiliations:** 1 Galvanize Therapeutics, Redwood City, CA, United States of America; 2 Hospital of the University of Pennsylvania, Philadelphia, PA, United States of America; National Research Council: Consiglio Nazionale delle Ricerche, ITALY

## Abstract

Chemo-immunotherapy uses combined systemic therapies for resectable and unresectable tumors. This approach is gaining clinical momentum, but survival increases leave considerable room for improvement. A novel form of Pulsed Electric Field (PEF) ablation combines focal tissue destruction with immune activation in preclinical settings. The PEFs induce lethal cell damage without requiring thermal processes, leaving cellular proteins intact. This affords PEF a favorable safety profile, improved antigenicity, and significant immunostimulatory damage-associated molecular pattern release compared to other focal therapies. Preclinical investigations demonstrate a combinatorial benefit of PEF with immunostimulation. This study evaluates whether this proprietary PEF therapy induces an immunostimulatory effect sufficient to augment systemic neoadjuvant chemotherapy and immunotherapy to reverse metastatic disease in an immune-cold murine tumor model. To determine whether PEF improves a neoadjuvant chemo-immunotherapy standard-of-care, partial PEF ablation was delivered to orthotopically inoculated 4T1 metastatic tumors in addition to combinations of cisplatin chemotherapy and/or αPD-1 immunotherapy, followed by resection. In addition, to determine whether PEF combined with chemo-immunotherapy improves local and metastatic response in unresectable populations, partial PEF ablation was added to chemo-immunotherapy in mice that did not receive resection. Blood cytokines and flow cytometry evaluated immune response. Partial PEF ablation generates an immunostimulatory tumor microenvironment, increases systemic immune cell populations, slows tumor growth, and prolongs survival relative to neoadjuvant systemic therapies-alone. These data suggest the addition of this proprietary PEF locoregional therapy may synergize with systemic standard-of-care paradigms to improve outcomes with potential or demonstrated metastatic disease in both resectable and unresectable patient cohorts.

## Introduction

Although cancer mortality rates have declined in the U.S. and other developed countries, the incidence of cancer continues to increase worldwide [1]. Improvements in patient survival may result from earlier screening and detection, new chemotherapeutic regimens, and targeted therapies. However, despite significant breakthroughs in the understanding, prevention, and treatment of cancer, the disease continues to affect millions of people worldwide.

One improvement in cancer therapy includes the introduction of systemic immunotherapies, which invoke immunostimulatory effects, empowering the immune system of patients to better locate and destroy cancer cells throughout the body. A particularly promising immunotherapy facet is the introduction of checkpoint inhibitors (CPIs) that block the immunoregulatory functions of CTLA-4, as well as the programmed cell death receptor-1 (PD-1) and its predominant ligand (PD-L1) [2]. While, several cancers have demonstrated significant therapeutic promise with the utilization of immune CPI therapy, a relatively small proportion of patients benefit from current CPI therapies [3, 4]. The low eligible population is mainly attributed to tumors described as immune “cold.” These tumors exhibit low levels of tumor-infiltrating lymphocytes (TILs), which are more likely exhausted, as well as insufficient tumor antigen availability and immunosuppressive microenvironments caused by tumor hypoxia and other mechanisms [5, 6].

Further improvements in standard-of-care paradigms include shifting from a resection-first approach to one that incorporates systemic therapies earlier in a neoadjuvant setting, followed by resection when possible [7, 8]. By delivering systemic therapies before surgery, delays systemic therapy administration for scheduling and recovery from major surgical procedures are eliminated, permitting systemic therapies to target micrometastases and circulating tumor cells earlier in the disease process while physically removing, irradiating, or ablating the primary tumor and visible metastases when feasible. At times, early induction of systemic therapies may result in tumor downstaging, permitting surgery in originally unresectable patients. Collectively, early delivery of systemic therapies has resulted in improvements in overall survival (OS) and disease-free survival (DFS) in patients relative to resection followed by adjuvant systemic therapy [9].

Pulsed electric field (PEF) therapy involves the delivery of brief electrical pulses to the targeted region to alter the native transmembrane potentials of cells and organelles. Some technologies, such as electrochemotherapy, use this effect to reversibly electroporate cells, improving macromolecule uptake to increase local drug toxicity following intratumoral or intravenous administration [10, 11]. Other technologies use a variety of downstream cellular effects, including sudden or accumulated injury to induce cellular demise via a myriad of cell death processes that are independent of thermal processes [12]. PEF for ablation may thereby be delivered in a manner that preserves stromal proteins comprising the extracellular matrix, preserving the functions of critical structures, such as the major vasculature, nerves, urethra, and pleural capsule [13, 14], offering a superior safety profile compared to thermal and irradiative ablation approaches as well as surgical resection [15].

In addition to improved safety profiles relative to other ablation, PEF cell death processes constitute an array of immunogenic cell death pathways, which release immunostimulatory cytokines, including damage-associated molecular patterns (DAMPs) and pathogen-associated molecular patterns (PAMPs) [16] while leaving tumor antigens intact. Aliya^TM^, a novel form of PEF, has been designed to optimize the particularly potent combination of DAMP release, antigenicity, and tumor microenvironment effects to offer improved upregulation of tumor-specific immune responses, which was previously shown preclinically to be superior to radiofrequency thermal ablation [17]. Preclinical investigations demonstrate this immune upregulation and improved outcomes for both immune “warm” CPI-responsive and immune “cold” CPI-unresponsive tumor models [18–22], and also demonstrated greater innate and adaptive immune cell populations relative to irreversible electroporation, another form of PEF ablation [23].

This study determined whether adding neoadjuvant PEF to standard-of-care systemic therapies can improve existing patient care paradigms. In particular, this novel form of PEF was added to a PD-1 neutralizing antibody (αPD-1) and cisplatin. PEFs comparable to those from a commercially available system were delivered to 4T1 tumors orthotopically implanted in the murine fat pad. This tumor model is an aggressive murine immune “cold” triple-negative breast cancer cell line with strong metastatic potential, where αPD-1 alone was previously shown to be ineffective [22]. The PEF dose used targeted ablating only 80% of the tumor volume to delineate the influence of immune system on tumor response. Some treatment groups underwent resection several days after the PEF treatment, representing a neoadjuvant therapeutic paradigm, while others left the tumor in situ to determine implications from the systemic therapy and partial-ablation alone. Mice were observed for primary tumor growth (if applicable), and blood was used to quantify cytokines and circulating immune cells to determine if the addition of PEF increased the anti-cancer immune response in a manner that complemented the systemic therapies accordingly.

## Materials and methods

### Study design

**Fig 1:**
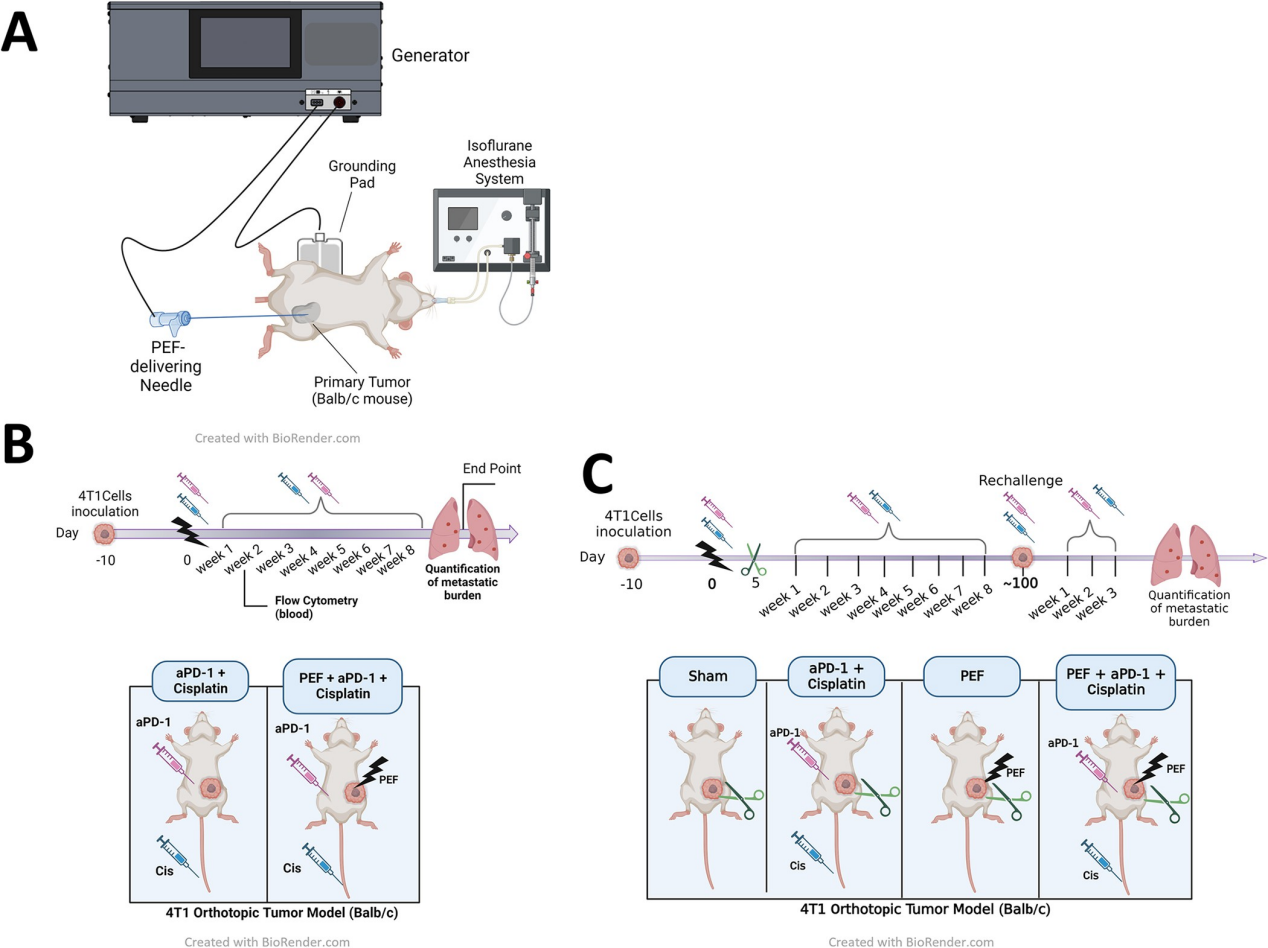
Study designs. A) 4T1 cells are inoculated in the 4^th^ fat mammary pad of Balb/c mice. 8–10 days later, a monopolar 25G needle is inserted in the tumor mass to deliver a single PEF treatment targeting approximately 80% of the tumor volume. A surgical grounding pad is placed on the shaved back of the mouse. Grounding pad and electrified needle are both connected to a modified Galvanize Aliya generator. B) Study 1 (unresected tumor) timeline: PEF is delivered at Day-0 (when tumors reached 5-7mm in the long dimension), and all mice began receiving αPD-1 (i.p) and cisplatin (i.v), with dosing repeated weekly for 8 weeks. Blood was collected 14 days post-treatment for flow cytometry analysis. Tumor growth and cancer burden was monitored until death or euthanasia, and lungs were collected for histological evaluation of metastatic burden from the mice that reached the endpoint. C) Study 2 (resected tumor) timeline: When tumors reached 5-7mm in the long dimension, mice were randomized into 4 treatment groups to receive sham, PEF-alone, Systemic Therapy-alone (αPD-1 and cisplatin), and the combination of PEF with Systemic Therapy. Primary tumors were resected on Day-5. Mice were monitored for up to ~100 days or euthanized due to extensive metastatic tumor burden. Surviving mice were reinoculated with 4T1 cells in the 2^nd^ mammary fat pad (rechallenge) as well as a naïve control group and all mice as well as a cohort of naïve controls were dosed with cisplatin and αPD-1 once weekly up to 4 total doses. A group of 5 naïve mice were inoculated with 4T1 cells but did not receive any systemic drug. Following reinoculation, the rechallenged tumors were measured until euthanasia when the lungs were collected for histological evaluation of metastatic burden.

The ability for this novel form of PEF therapy to enhance local and systemic reduction in tumor burden and metastatic disease was investigated over two separate studies, each addressing different clinical archetypes. All studies were performed in accordance with the Institutional Animal Care and Use Committee (IACUC) of Bayside Biosciences (Santa Clara, CA) covered by the protocol number 2023-05-01. An array of treatment paradigms were investigated in each, and different endpoints were extracted (Fig 1B and 1C).

The first study (Fig 1B) investigated the benefit of adding this novel PEF to standard systemic therapy conditions in metastatic disease with unresectable primary tumors. Whereas the comparison related to improving standard-of-care (SOC) therapies, only two groups were included: a systemic-only combination therapy (cisplatin + αPD-1) and one that added partial PEF ablation to systemic therapy (PEF + Cisplatin + αPD-1) (Table 1: Groups 1 and 2, respectively). Primary tumor growth was measured thrice weekly using digital calipers. Mice bearing tumors that exceeded 1500 mm^3^ or demonstrating excessive metastatic burden (e.g., weight loss > 15%, lethargy) were euthanized. At the end of the study, the surviving mice were euthanized to check for lung metastases.

**Table 1:** Treatment groups for each study.

Group #	Group Name	IgG	αPD1	Cisplatin	PEF	Resection	n
1	αPD-1 + Cisplatin (no resection)	-	+	+	-	-	10
2	PEF + αPD-1 + Cisplatin (no resection)	-	+	+	+	-	10
3	Sham	+	-	-	-	+	10
4	αPD-1-only	-	+	-	-	+	10
5	Cisplatin-only	-	-	+	-	+	10
6	αPD-1 + Cisplatin (SOC*)	-	+	+	-	+	10
7	PEF-only	-	-	-	+	+	10
8	PEF + αPD-1	-	+	-	+	+	10
9	PEF + Cisplatin	-	-	+	+	+	10
10	PEF + αPD-1 + Cisplatin	-	+	+	+	+	10

*SOC = Standard of care

The second investigation (Fig 1C) determined whether adding neoadjuvant PEF to different treatment paradigms could reverse the occurrence of metastatic disease (“abscopal effect”) in clinical scenarios with resectable tumors. Once tumors reached the appropriate size, they were treated with various treatment iterations (Table 1: Groups 3–10). Notably, a prior investigation confirmed the pre-existence of metastatic disease in the 4T1 tumor by the treatment day (Day-0) [24]. Surgical removal of the inoculated tumor was performed on Day-5 to all mice. This mimics surgical resection in patients where the cancer has spread to the rest of the body, especially Stage III and Stage IV cancers, where distant recurrence may occur despite removal of all radiographically evident disease. Following resection on day 5, the overall health and survival of the mice were monitored for death or euthanasia due to metastatic burden (e.g., weight loss > 15%, lethargy). On Day 104, surviving mice received a late-stage rechallenge via reinoculation in the 2^nd^ mammary fat pad (axilla) to evaluate the durability and longevity of any tumor-specific immune response. As a control, ten naïve mice were inoculated at the same axillary fat pad location. On the same day of reinoculation, systemic therapy (cisplatin + αPD-1) was restarted in the surviving mice. Systemic therapy was also initiated in five naïve inoculated mice to determine de novo benefits of the therapy to mice with new inoculations. Mice were euthanized 26 days post-inoculation, and the presence of lung metastases was evaluated histologically and quantified.

#### Mouse tumor model

4T1, a triple-negative breast cancer cell line procured from the American Type Culture Collection (CRL-2536; Manassas, VA, USA), was authenticated through short Tandem Repeat analysis and used for all *in vivo* experiments. The cells were cultured in RPMI 1640 medium (MT10040CV, Corning, Manassas, VA, USA) containing 10% fetal bovine serum (092910154, MP Biomedicals, Irvine, CA, USA) and 1% Antibiotic-Antimycotic (15240–062, Gibco Life Technologies Corporation, Grand Island, NY, USA) at 37°C and 5% CO_2_.

A total of 200,000 4T1 cells (resuspended in PBS) were inoculated into the mammary fat pad of 4-to 6-week-old BALB/c mice (Charles River Laboratories, Boson, MA) to generate an orthotopic tumor in the mammary gland. Mice were housed together in ventilated cages (4–6 mice per cage) subject to light dark cycles. Food and water were provided *ad libitum*. Food, water, and bedding were changed and/or replaced two times per week.

The well-being of mice was observed daily, and when an end point was reached, euthanasia was administered by CO_2_ asphyxiation immediately or within a few hours. Criteria for euthanasia included a tumor exceeding 1.5cm in any direction, tumor preventing ambulation or ability to reach food and water for more than 24 hours, if tumors became severely ulcerated or abscessed, if mice became emaciated or lost ≥ 20% body weight, or if the mice showed signs of lethargy and labored breathing. If unexpected death unrelated to tumor burden occurred, the animal was excluded from data analysis.

*Interventions*. Once the tumors reached 5–7 mm in size (10 days after inoculation), the mice were randomly assigned by cage number to the experimental groups (Table 1) and interventions began (Day-0). On Day 0, mice allocated to groups with PEF (2, 4, 6, 7, 8, and 10) underwent PEF treatment delivery into the center of the tumor. On Day 0, groups 2, 3, 5, 6, 7, 8, 9, and 10 started weekly intravenous cisplatin (2 mg/kg) and/or intraperitoneal αPD-1 (200μg, i.p) [25]. Systemic treatments were administered once a week for eight weeks. Tumors were monitored and measured with electronic calipers three times per week, and tumor volumes were calculated according to the following formula: Tumor Volume = (long dimension x short dimension) x (short dimension/2).

Endpoint criteria for euthanasia included a maximal tumor volume greater than 1500 mm^3^ or indications of excessive metastatic tumor burden, including weight loss (> 15%) and lethargy.

### Drugs and dosing

#### Cisplatin toxicity

A pilot study was performed to determine the toxicity threshold of systemic cisplatin. Forty 6–8-week-old female mice were dosed intravenously in the tail vein once per week for 14 weeks with 1, 2, 4, and 8 mg/kg cisplatin (SC200896, Santa Cruz Biotechnology Inc., Dallas, TX, USA) resuspended in PBS. Cisplatin doses causing >15% body weight loss were not considered in future studies. For the experimental protocol, cisplatin was resuspended in PBS and administered intravenously into the tail vein at a dose of 2 mg/kg (100 μL volume). Mouse weight was monitored three times per week. Mice with more than 15% body weight loss were euthanized.

#### αPD-1 and IgG

The monoclonal antibody αPD-1 and isotype control IgG were purchased from BioXCell (BE0146 and BE0089, Lebanon, NH, USA) and administered intraperitoneally (i.p.) at 200 μg per injection (100μl volume of 2μg/μl diluted in PBS). During the resection and non-resection studies, antibodies were administered once per week for 8 weeks. In the rechallenge study, the mice were dosed once per week for 4 weeks.

### Pulsed Electric Field (PEF) system

The PEF system used in this study comprised a titrated version of the waveform applicable to Aliya^TM^ (Galvanize Therapeutics, CA, USA). This PEF system delivers a series of biphasic PEFs in a monopolar configuration, where a single needle electrode delivers energy to the targeted tissue with a distant dispersive return electrode.

#### PEF partial treatment dose determination

Partial ablation of the inoculated tumors is necessary to properly delineate the benefits of the induced immune system on local tumor response. Thus, prior to initiating this study a series of pilot studies were performed to determine an appropriate dose that would ablate approximately 80% of the total tumor volume, as described in [24]. Briefly, PEF was delivered at varying voltage intensities to tumors measuring 5–7 mm in the long dimension. Mice were euthanized at 3–4 days post-PEF, and histological analysis with serial sectioning and H&E staining was used to measure the area of ablation relative to the tumor. The final dose selected was noted to ablate approximately 80% of the tumor volume, characterized by a central zone of cell death, with a periphery of viable cancer cells.

#### PEF treatment delivery

Once tumors reached approximately 5 mm in diameter in the short dimension (Day-0), PEF was administered to the tumors in the applicable groups. To deliver PEF, mice were anesthetized with isofluorane (3% induction, 2% maintenance) and O_2_ inhalation using a chamber to induce anesthesia, and a nose cone for maintenance during the procedure. Mice were shaved and placed supine on a dispersive electrode (3M universal electrosurgical pad, cat#9165E, Saint Paul, MN, USA). A custom-built 25-gauge needle (5.0 mm long electrical exposure) was inserted through the skin and centered in the tumor. Following needle placement, a PEF treatment was delivered for approximately 5 min. A schematic representation of the system is shown in Fig 1A. Following PEF delivery, the needle was removed and mice were recovered, and a 1ml bolus of 0.9% saline was administered subcutaneously.

### Flow cytometry

Flow cytometry was used to quantify systemic immune cell populations at the timepoints depicted in Fig 1. Approximately 200 μL of blood was collected from groups 1 and 2 in EDTA-containing tubes on day 14 (Table 1). Red blood cells were lysed using 1 ml of red blood cells Lysis buffer (420201, Bio-Legend, San Diego, CA, USA). Blood was mixed briefly to resuspend the remaining cells (peripheral blood mononuclear cells, PBMCs), incubated for 15 min at room temperature, and centrifuged. The PBMCs were resuspended in FACS buffer (B51503, Beckman Coulter, Brea, CA) and incubated in Fc blocking buffer (156604, anti-mouse CD16/32 antibody, BioLegend, San Diego, CA, USA) at room temperature for 15 min. Next, the cells were divided into individual tubes for the respective cell type analyses, suspended in 50 μL of staining buffer (420201, BioLegend, San Diego, CA, USA), and stained for the designated cell type. The antibodies used are listed in S1 Table. After staining, the samples were washed and fixed with 1% paraformaldehyde (J61899; Alfa Aesar, Ward Hill, MA, USA) before analysis using a CytoFLEX3 flow cytometer (Beckman Coulter Life Sciences, Brea, CA, USA).

In addition, a 4T1 antigen-specific tetramer (Gp70) was used to quantify the generation of tumor-specific CD8+ T cells (TB-M521-1, Mbl International, Woburn, MA, USA). Approximately 200 μL of total blood was incubated with tetramer gp70-PE and CD8+a-BV421, according to the manufacturer’s instructions. Flow cytometry was performed to quantify the number of tetramer-positive CD8+ lymphocytes.

Raw data were analyzed using Kaluza software (Beckman Coulter, San Jose, CA, USA).

### H&E

Tumor and lung samples were collected after euthanasia, fixed in 10% formalin for 24 h, and embedded in paraffin blocks. Tissue sections of 5 μm thickness were cut with a cryotome and stained with hematoxylin and eosin (H&E) at Histo-Tec (Hayward, CA, USA). Sections were produced by cutting tissue block every 200μm.

### Serum cytokines analysis

Blood samples collected on day 10 from mice in groups 1 and 2 (Table 1) were left to clot for 30 min at room temperature and then centrifuged at 1000*g* for 10 min in a refrigerated centrifuge. The resulting supernatant, designated as serum, was sent to Eve Technologies (Calgary, Canada) for quantification of 44 cytokines (S2 Table).

### Statistical analysis

All statistical analyses were performed using the GraphPad Prism software (GraphPad Software Inc., San Diego, CA, USA). The statistical significance of the Kaplan-Meier curves was calculated using the log-rank test. The difference between the means of unpaired samples was calculated using two-way analysis of variance (ANOVA) and Student’s t-test. Tumor growth kinetics were compared using ANOVA. P-values ≤0.05 are reported in the graphs.

### Data integrity

To minimize bias, data were managed by third parties and blinded as-appropriate. Mice were randomly assigned to treatment groups by cage number. An independent contract research organization (CRO) managed mouse husbandry, performed tumor inoculations and tumor measurements, managed euthanasia decisions, administered αPD-1 immunotherapy, and collected all biological samples (tumor, blood, and lungs) for analysis. Additional CROs performed histopathological processing and cytokine quantification. All CROs were blinded to mouse treatment conditions apart from administration of systemic therapies, as needed. Flow cytometry was performed identically on all mice with a standard gating strategy (S5 Fig). Metastasis confirmation and quantification was performed by authors using ImageJ.

## Results

### Cisplatin toxicity

Fourteen doses of cisplatin were administered over the course of 14 weeks at doses of 1, 2, 4, and 8 mg/kg, and the mice were observed for up to 144 days. The 8 and 4 mg/kg doses of cisplatin induced body weight loss that required euthanasia of all mice in the groups on days 28 and 63, respectively. No weight loss was observed during the study period for either the 1 or 2 mg/kg doses of cisplatin, indicating that the 2 mg/kg dose used in this study was well tolerated (S1 Fig).

### Partial PEF ablation dose confirmation

The pilot studies to determine appropriate PEF dose for targeting approximately 80% ablation of the total tumor volume determined an appropriately titrated dose. H&E staining of tumor samples collected 3–4 days post-PEF at this dose show a central zone of decellularized and dying cells corresponding to the area where the energy was deposited, surrounded by a rim of viable tumor cells, confirming the partial ablation of the targeted tumors (S2A and S2B Fig).

### Adding PEF to standard-of-care cisplatin and αPD-1 provides local and systemic benefits, prolonging the survival of mice bearing tumor

**Fig 2:**
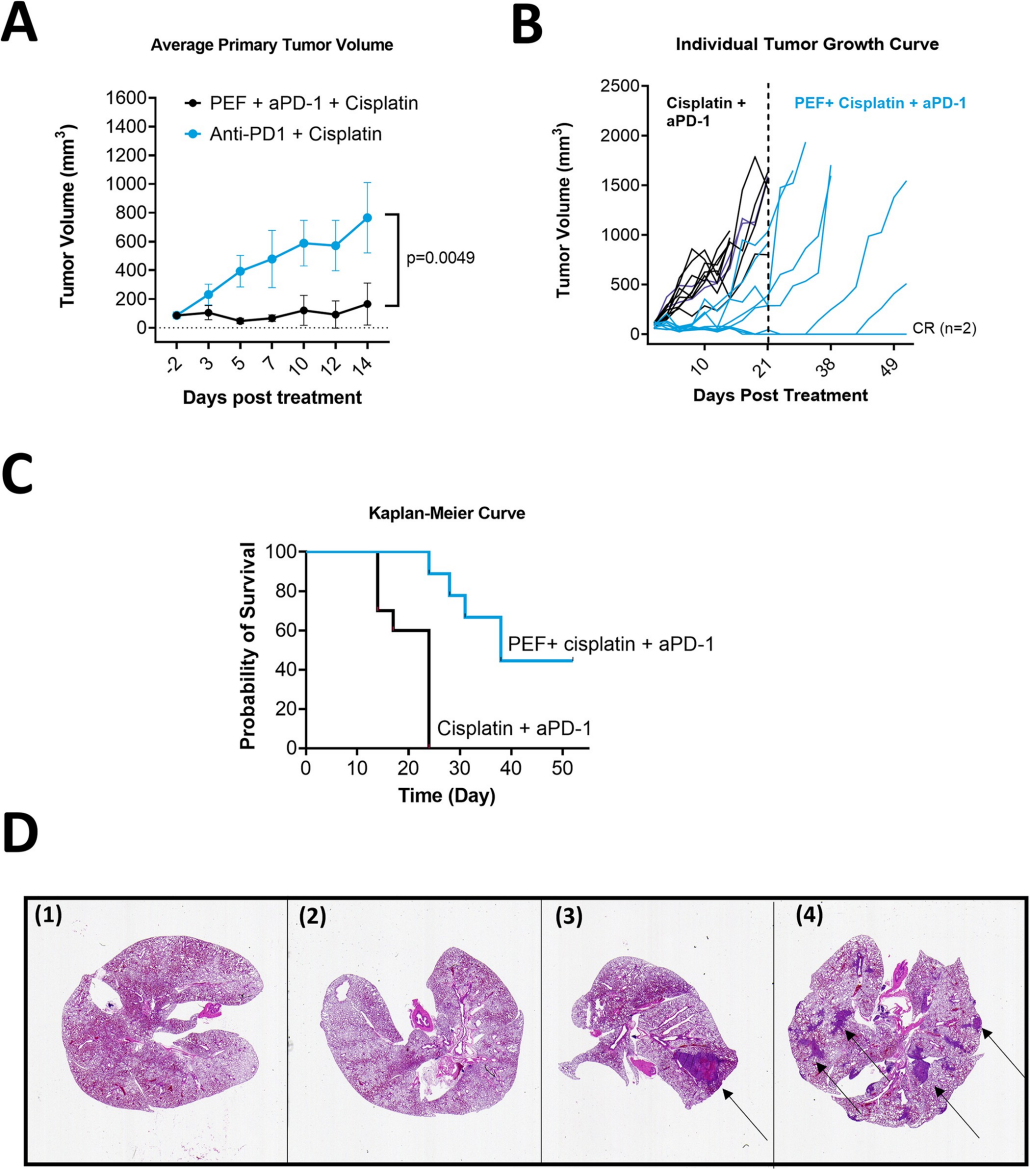
Treatment response in mice without tumor resection. A) The graph reports the tumor growth curve of unresected groups up to Day-14 before any mice mortality. B) Individual tumor volume for each mouse in the respective group until the day termination of the study (Day-52). Two mice (2/10) in the PEF+ cisplatin + αPD-1 achieved complete response (CR). C) The Kaplan- Meier curve indicates mouse survival the cisplatin + αPD-1 systemic therapy paradigm had 0% of mice alive at Day-24, and 44% alive at Day-52 when PEF was added to the systemic therapy. D) Lungs histology on Day-52 from the 4 surviving mice show representative tissue sections that captured the most metastases. Panels D(1) and D(2) show the absence of metastases in the two mice with complete primary tumor response. Panel D(3) shows one single large metastasis (black arrow), and Panel D(4) shows multiple metastases (black arrows) in the remaining mice that survived to the terminal endpoint.

To investigate the benefit of adding PEF to chemotherapy and immunotherapy in local tumor control, inoculated tumors remained in situ after ablation or sham treatment throughout the study duration. Two therapeutic groups, either systemic therapy-alone (cisplatin + αPD-1) or systemic therapy with PEF ablation of the primary tumor (PEF + Cisplatin + αPD-1), were compared. One mouse in the PEF with systemic therapy group was erroneously euthanized on Day 31 by the CRO and excluded from survival and tumor volume analysis (Fig 2B and 2C). Systemic-only therapy mice exhibited faster tumor growth than those in the group that also received PEF partial tumor ablation (Fig 2A). All systemic-only therapy mice required euthanasia due to excessive primary tumor size by day 24 (Fig 2B and 2C). However adding PEF to the systemic therapy regimen significantly improved survival, with 44% of mice surviving to the 55-day endpoint (log-rank p = 0.0002) (Fig 2C). Furthermore, two mice in the PEF+systemic therapy group showed a complete response via regression of the primary tumor and an absence of histologically visible lung metastases (Fig 2D). This demonstrates that adding partial PEF ablation to systemic therapy combinations improves primary tumor control and also facilitates a systemic abscopal effect not observed in systemic-combinations alone.

### Pulsed electric field therapy improves circulating immune cell profiles

**Fig 3:**
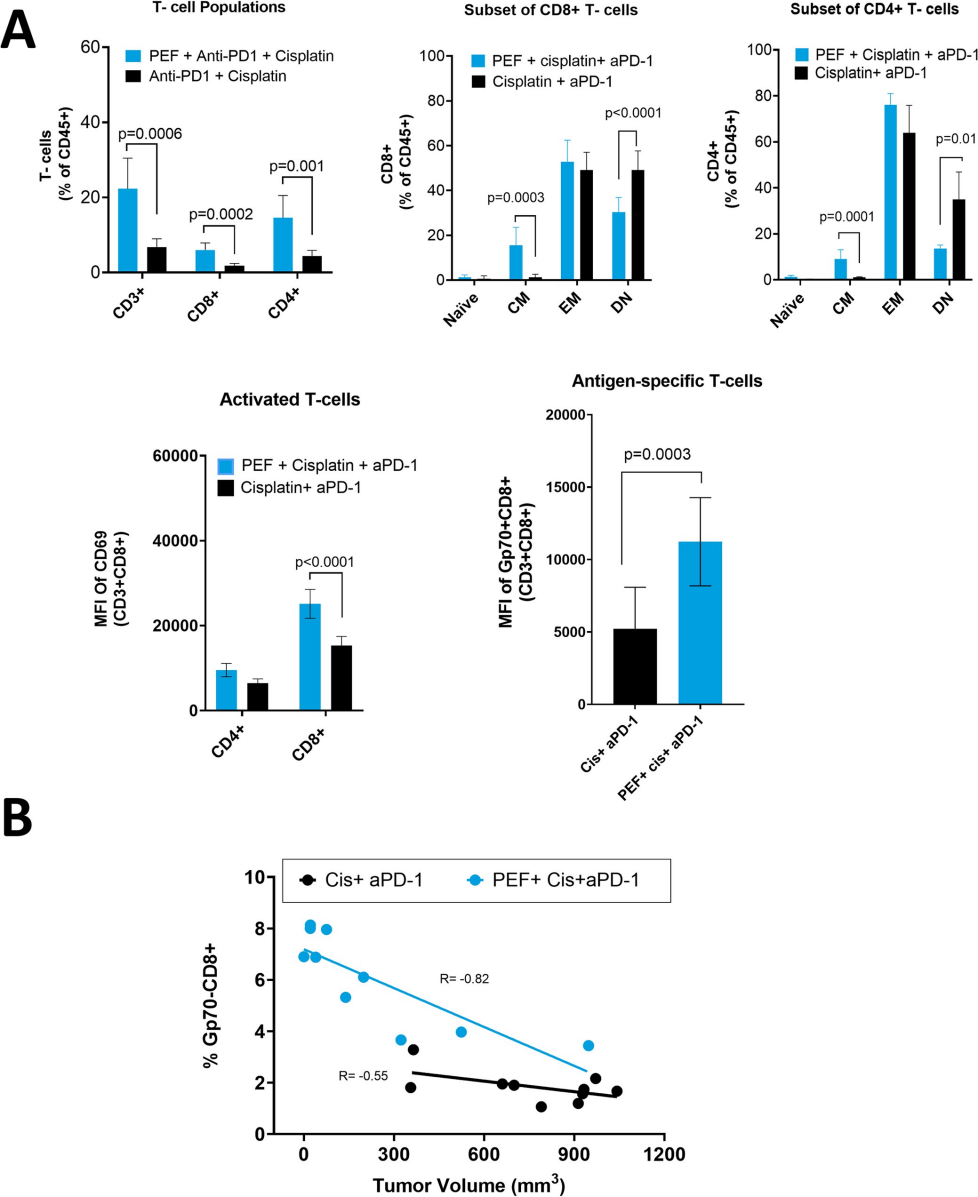
Flow cytometry immune profiles in mice without resection. A) Histograms of different T-cell populations (CD3+, CD4+, CD8+) and subpopulations, including (top row) Naïve T-cells, Central Memory (CM), Effector Memory (EM) and double negative (DN) within the CD4+ and CD8+ populations. (Bottom left) Mean fluorescence intensity (MFI) for CD69+ (activated) CD4+ and CD8+ T-cells. (Bottom right) MFI of antigen-specific tetramer conjugated to CD8+ cells. B) The correlation between Day-14 ttumor volume and circulating CD8+ T-cells. The Pearson’s correlation coefficient (R) is reported in the graph.

Representative dot plots for the flow cytometry and gating strategy are provided in S5 Fig. Circulating immunocyte analysis performed on day 14 in mice without resection (Table 1: Groups 1 and 2) demonstrated a statistically significant 3- to 3.6- fold increase in CD3+, CD4+, and CD8+ T-cells when PEF was added to systemic therapy relative to systemic therapy alone (Fig 3A). Subpopulations of CD8+ T cells were determined via activation and exhaustion markers. CD8+ T-cells in the PEF-inclusive group displayed significantly elevated levels of the activation marker CD69 (measured by MFI) compared to the systemic-only therapy group (Fig 3A). Moreover, there was an increase in both CD4+ and CD8+ Central Memory (CM) T-cells (CD44^high^, CD62L^high^) when PEF was added to systemic therapy group compared systemic therapy alone. Double negative (DN) (CD44^low^CD62L^low^) CD4+ and CD8+ T-cell subpopulations were decreased in the mice with PEF added, while there were no differences in naïve CD8+ or CD4+ T cells (CD44^low^, CD62L^high^) between the treatment groups (Fig 3A). Tetramer staining for endogenous tumor-associated gp70 demonstrated a significant 2-fold increase in gp70+/CD8+ T-cells when PEF was added to systemic therapies (cisplatin + αPD-1) relative to systemic therapies alone **(**Fig 3A**)**.

The Day-14 percentage of circulating CD8+ cells was positively correlated with tumor growth reduction, demonstrating that immune cell population changes were related to primary tumor response (Fig 3B). This relationship was stronger when PEF was added to systemic therapy (Pearson Correlation coefficient R = -0.8, R^2^ = 0.67), than for systemic-therapy only (R = -0.55, R^2^ = 0.30).

### Adding PEF to SOC systemic therapy decreased pro-tumorigenic cytokines

Forty-four cytokines were profiled on day 10 in the serum of mice that did not undergo tumor resection (Table 1, Groups 1 and 2). There were statistically significant changes in 17 cytokines, indicating a major impact of PEF relative to systemic therapy administered alone (S3A Fig). Among these, pro-tumorigenic IL-5, IL-13, and CCL5 (RANTES) were reduced by 1.9- to 3-fold in PEF-treated mice compared to the group that received only systemic therapy (S3B Fig). Moreover, vascular endothelial growth factor (VEGF) was reduced by 1.9-fold in the PEF group. Macrophage- and DC-associated chemokines such as MIP-1a, MIP-1b, MIP-3b, GM-CSF, and G-CSF were also significantly reduced. Moreover, there was an increase in neutrophil and NK cell chemotactic mediators (S3A Fig). Importantly, at the time point of blood draw for cytokine analyses, the average tumor volumes of the systemic-only and PEF + systemic therapies were 537 versus 120 mm^3^, respectively.

### Primary tumor resection study

#### Primary tumor growth before resection

**Fig 4:**
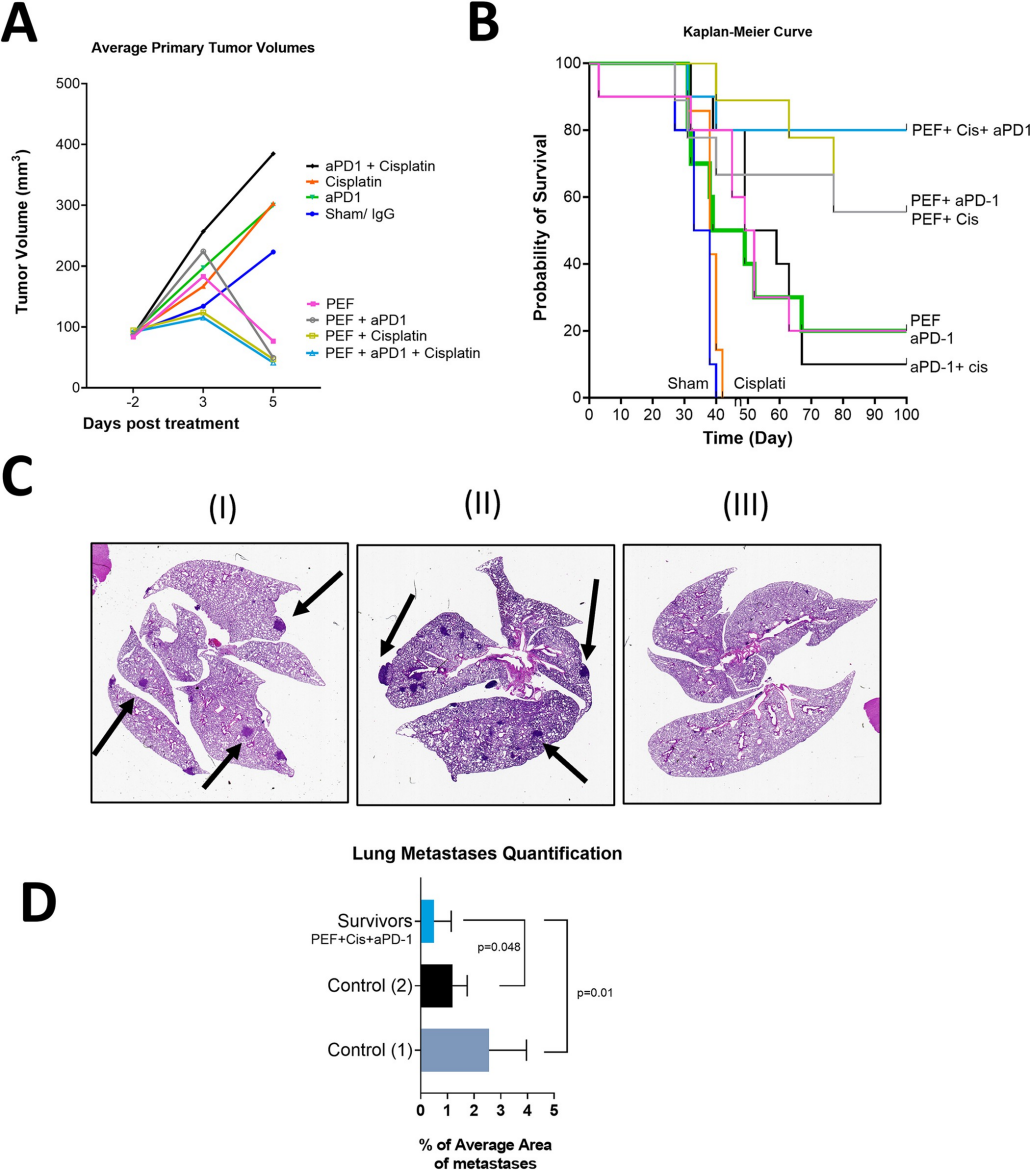
Survival curve and abscopal effects from treat-and-resect study. A) Average tumor volume for each experimental group until the day of resection (Day-5). B) Kaplan-Meier survival data resulting from spontaneous death or euthanasia due to extensive metastatic burden. C) Representative lung section stained with H&E from untreated control that received systemic therapy (I), untreated control mice (II), and survivor from the PEF+ systemic therapy (III). Representative clusters of metastatic cells are indicated by black arrows. D) Histological area of metastases as percentage of total lung area across serially-sectioned lungs.

Tumor growth curves for the eight treatment conditions prior to resection are provided in Fig 4A. All systemic-only groups demonstrated progressive tumor growth. All groups that included partial PEF ablation had short-term volmue increase likely reflecting edema and immune cell infiltration post-ablation, which was followed by a reduction in tumor size by Day-5 (resection timepoint). Tumor measurements prior to resection are provided in S3 Table. Notably, mice receiving systemic-only therapies received a single dose on Day-0, which likely did not have sufficient time to alter tumor growth.

#### Adding PEF to SOC systemic therapy improves survival in resected mice

Following resection, the mice were monitored until spontaneous death or euthanasia due to extensive metastatic burden including lethargy or weight loss (>15% of body weight). Mice with recurrent primary tumor growth suggested incomplete resection and were excluded from the survival analysis (one from Group 8, one from Group 9, and three from Group 5). The Kaplan-Meier survival curve (Fig 4B) demonstrates that adding partial PEF ablation improved survival relative to any systemic therapy condition, with the 100-day survival of the PEF-alone group at 20%, the addition of PEF to either systemic monotherapy (cisplatin or αPD-1) reaching 55%, and adding PEF to the standard-of-care surrogate (PEF+ cisplatin+ αPD-1) attaining 90% survival. Because no mice included in this analysis died from primary tumor burden, all data suggest survival resulted from elimination of systemic cancer burden.

#### PEF treatment in conjunction with systemic therapy offers better immunity after tumor rechallenge

All mice that survived for 104 days were restarted with systemic therapy and rechallenged with 4T1 cells, along with naïve control mice, as defined in the Methods section. All control groups and the survivors from the previous treat-and-resect study developed tumors (S4 Fig). Differences in metastatic burden were determined by euthanizing all surviving mice 26 days post-rechallenge. As expected, significant lung metastatic burden was noted in the control group mice as well as in the previously treated mice (survivor), with the exception of the group that had received PEF+ cisplatin+ αPD-1 (Fig 4C). In the latter case, the metastases were either absent or very small. Metastatic area quantification of histological sections demonstrated that PEF + cisplatin + αPD-1 survivor mice had metastases covering 0.49% of the lung surface area statistically lower than the untreated Control (1) group and Control (2) group that received cisplatin+αPD-1 which presented metastases in 2.5% and 1.19% of the lung surface (p = 0.01 and 0.048 respectively) (Fig 4D). All raw data are available in S4 Table.

## Discussion

This study evaluates the ability for a new form of pulsed electric field (PEF) treatment to improve local and systemic outcomes in a challenging orthotopically implanted murine breast cancer tumor model when used in combination with common systemic therapy clinical paradigms. The 4T1 model is a triple-negative breast cancer and is commonly regarded as immune “cold,” [26] providing a challenging target to determine whether PEF can provide synergistic resensitization to immunotherapy. In one experiment, the primary tumor remained in situ and partial PEF ablation was delivered to determine whether it would improve primary and distant responses. In a separate experiment, resection of the primary tumor several days after partial PEF ablation was used to determine whether adding PEF to systemic therapies invoked supplementary off-target treatment effects and metastatic clearance.

Importantly, in a similar study performed prior to the one described here, an entire cohort of untreated mice with the same tumor model resected on Day-0 died by day 44 (n = 10) [24], confirming that in these studies micrometastases were present prior to any treatment, with 100% penetrance. This suggests that any subsequent absence of metastases likely reflects the clearance of pre-existing metastases, rather than simply preventing metastasis-shedding by the primary tumor after treatment.

In this study, the addition of PEF to systemic therapy (cisplatin + αPD-1) inhibited primary tumor growth and improved mouse survival when the tumors were not excised. Favorable cytokine profiles and systemic immune cell population upregulation on Day-14 in the group with PEF added correlated with the primary tumor response. In the groups that underwent resection on Day- 5 post-treatment, partial PEF ablation-alone improved survival from metastatic burden beyond resection alone, suggesting systemic immune activation. Furthermore, when PEF was added to either systemic therapy, survival markedly improved beyond systemic therapy alone. Finally, when PEF was added to the standard-of-care surrogate for neoadjuvant treatment (cisplatin + αPD-1, followed by resection), 80% survival was obtained, far superior to the 10% survival post-resection from metastatic protection afforded by the neoadjuvant chemotherapy and immunotherapy systemic therapy combination. Further, the lungs of mice that had previously cleared their tumors in the setting with PEF had statistically fewer and smaller metastases than those from the control groups, demonstrating a degree of durable immune control of this aggressive tumor cell line.

For the resection study, previously published data demonstrated that metastases existed by treatment Day-0 in the 4T1 tumor model [24]. This was confirmed with mice that underwent surgical resection after tumors reached the typical size for initiating treatment (Day-0). No intervention was administered to this cohort, and mice were monitored until euthanasia or spontaneous death, where 100% of the mice died by Day 44, confirming the -pre-existence of systemic disease by Day-0. Visual inspection and histology of the lungs were performed to confirm a correlation with the cause of death to metastatic spread.

The data here suggests a synergistic effect, whereby the inclusion of partial PEF ablation to systemic therapy treatment paradigms induces primary tumor response improvements while activating innate and adaptive immune responses. Importantly, this provides strong evidence that the addition of targeted PEF ablation can produce systemic improvements and abscopal effects, preventing or clearing distant metastases via activation of the immune system, which is further bolstered by the addition of systemic chemotherapy and checkpoint blockade (S6 Fig**)**.

Cytokine profiling conducted in the serum of mice that did not undergo resection 10 days post-ablation indicates that adding PEF to systemic therapy resulted in a general reduction in mediators associated with macrophage and dendritic cell (DC) chemotaxis (MIP-1a, MIP-1b, MIP-3b, GM-CSF, and G-CSF), possibly reflecting a reduced number of macrophages and DC in and around the tumor. Moreover, an increase in neutrophil and natural killer cell (NK) chemotactic mediators was noted. In mice that received PEF, the increase in LIX and Fractalkine would support more migrating neutrophils and NK cells, suggesting more systemic pro-inflammatory signaling, which could be anticipated to be antitumor. Additionally, several pro-tumorigenic cytokines, such as IL-5, IL-13, and CCL5, were found to be strongly downregulated in the group that added PEF relative to the systemic therapy-alone group. The analysis of circulating cytokines in the unresected cohort revealed that VEGF protein was significantly downregulated in the serum of mice that received PEF in addition to systemic therapy. VEGF promotes angiogenesis, and elevated levels of VEGF in blood have been associated with poor prognosis in several types of cancer, including breast, colorectal, lung, and ovarian cancers [27, 28].

Partial ablation of PEF was used to provide appropriate sensitivity for tumor response evaluation, where total ablation could potentially clear the primary tumors in the unresected groups, obscuring the role of the immune system in attaining total tumor clearance. However, it has been hypothesized that additional treatment zone size, immunostimulatory DAMP, and antigen release may produce superior immune responses equivalent to those observed here. Future studies should explore whether total ablation further improves abscopal response in immune “cold” as well as immune “hot” tumor models. Notably, literature has shown that thermal ablation methods, such as radiofrequency (RF) ablation, induce counterproductive responses in partial ablation experimental mouse models, where partial ablation induced stronger primary tumor growth and greater metastatic potential of the treated tumor [29–31] suggesting a significant risk when patients receive incomplete or discontinuous RF ablation. Comparably, partial PEF ablation was not observed to incur these effects here, consistent with [24], which demonstrated PEF partial ablation invoked superior immunostimulation, tumor response, and mouse survival relative to RF ablation.

A low cisplatin concentration (2 mg/kg) was selected to represent modest doses if clinically-translated. The combination of cisplatin administration with PEF used here was designed to simulate adoption of PEF delivery into typical patient care paradigms that involve multiple chemotherapy doses. It should be noted that this approach is distinct from combinatorial approaches that rely on the combination to evoke their inherent effect, such as electrochemotherapy (ECT) [10]. In ECT, a chemotherapeutic (generally bleomycin or cisplatin) is delivered intratumorally or systemically, and while it is in modest concentrations, a reversible electroporation protocol (generally 8 monophasic pulses of 100 μs duration) is delivered with a bipolar electrode array to the targeted site, reversibly increasing cellular permeability to the interstitial agent, resulting in a dramatic increase in cellular uptake of the chemotherapy in the reversibly electroporated tissue near the electrodes used. For this approach to work, the electroporation pulses must be delivered when drug concentrations are optimal at the targeted site, which is generally done approximately seven minutes after drug injection [10, 11].

Conversely, the approach used here delivered chemotherapy without regard to the timing of PEF delivery, and subsequent cisplatin injections were performed without any PEF delivery. In these models, the PEF delivered was sufficient to induce ablation of the mouse tumors, as indicated in S2 Fig. Thus, the combinatorial approach used here relies more on additive effects of combining existing therapy protocols. Future work may explore the mechanisms underpinning the cellular and tissue-scale changes that occur when the various combinations are used to discern if the cisplatin is specifically invoking additional immunostimulatory effects on the cells it destroys, and whether this effect is unique to cisplatin, or would be possible with other chemotherapeutics. Furthermore, tthe enhanced efficacy from adding PEF to the low-dose chemotherapy regimen used in this study also suggests that adding PEF to systemic cancer treatment paradigms may potentially afford patient improvement with lower systemic chemotherapy doses, offering improved morbidity profiles and increasing the addressable patient population that cannot tolerate typical chemotherapy regimens.

This study demonstrated an additive benefit of PEF treatment in mice treated with both resected and unresected tumors. This affords promising translational potential, where a broad spectrum of patient and tumor conditions exist, from Stage I readily resectable cases to Stage IV metastatic disease. When patients anywhere along this spectrum receive systemic chemotherapy or immunotherapy as part of their treatment paradigm, it is thus likely that the inclusion of PEF therapy will further improve their outcomes. The ability to employ PEF ablation in a neoadjuvant setting results from its superior safety profile and sparing of the extracellular matrix [32] (*data under review*) which permits safe subsequent surgical resection, a characteristic unique from other focal therapies.

The safety profile and immunostimulatory characteristics demonstrated here suggest that a broad range of patients may benefit from PEF ablation. Relative to surgical resection or other targeted therapies, these murine data show PEF ablation can destroy the targeted primary tumor with less risk and may bolster the immune system to invoke a systemic protective benefit against any circulating tumor cells or micrometastases. In addition, the use of the novel single-needle monopolar PEF system enables simplified clinical procedures compared to PEF technologies that use bipolar electrode arrays, reducing the barrier-to-entry for treatment. Collectively, these characteristics raise the possibility of using PEF technology as a first-line therapy to induce targeted ablation and initiate a systemic immune response at the time of biopsy cancer confirmation, which is followed by subsequent standard-of-care treatment paradigms.

The role of PEF in modulating the immune system has been the focus of numerous studies in recent years [18, 19, 33–35], and recent literature has demonstrated a spectrum of cell death mechanisms from PEF ablation, which shift based on the characteristic waveform. Here, we present immunological and tumor response findings for a representative biphasic waveform delivered through a single needle electrode (monopolar configuration). Importantly, the Aliya PEF system used here uses prescribed, versus variable, primary and secondary electrical parameters, ensuring accurate translation of the parameters used here minus the titrated voltage to target 80% tumor volume. It is possible that the other cell death mechanisms for other PEF ablation technologies may have differential effects on the immune system, which may not produce the same additive benefit as the systemic therapies demonstrated here.

One key to interpreting the data from this study was the chronology of metastases in the 4T1 model. It was shown that, at the time of treatment initiation (Day-0), mice already had circulating tumor cells and/or metastases, as evidenced by a cohort of mice require their euthanasia due to metastatic burden despite Day-0 resection. Thus, as the intervention was initiated, metastases were already present, which were later cleared by the immune response, offering a systemic immune abscopal effect on distant diseases invoked by PEF. Furthermore, with deliberate partial ablation, viable tumor cells remained *in situ*, which did not proliferate, as noted by the considerable lung and brain metastases in the systemic-therapy-only mice, whereas the addition of PEF to systemic therapy had significantly attenuated or completely absent metastases in these organs.

There are several limitations to this study. Firstly, the objective of this study was to determine whether the addition of PEF to existing patient care protocols could improve their outcome. Because clinical standard-of-care continues to progress towards combinatorial chemotherapy and immunotherapy [36] a granular separation of all potential treatment iterations was not included in the unresected study. Further, data relating to individual contributions was previously reported in [24], which showed a degree of distant tumor clearance due to PEF-induced immunostimulation, but which was significantly bolstered by combinatorial approach. Another limitation is the use of partial PEF ablation to delineate the benefit of immune response on local tumor control, which may not represent clinical scenarios, where total tumor coverage plus margin is targeted. Future studies should determine optimal ablation methods to generate the greatest systemic immunostimulatory benefit.

## Conclusion

This study delivered a proprietary form of PEF ablation to a challenging, triple-negative immune “cold” orthotopically implanted murine tumor model to determine whether the addition of PEF provides synergistic local and distant benefits to conventional systemic therapies, including chemotherapy and immunotherapy. It found that the addition of PEF evoked systemic immune cell upregulation, ultimately resulting in improved survival via elimination of metastases in mice that underwent resection. Systemic cytokine profiling and flow cytometry demonstrated an increase in the tumor-specific immune response. In mice that did not undergo resection, PEF produced improved local tumor response over combined systemic-only therapy, as well as reduced presence of metastases, including a number of mice that achieved total tumor clearance. In mice with tumors subsequently resected, PEF ablation prior to resection improved survival for all systemic therapy combinations evaluated. These data suggest an independent and additive benefit for incorporating PEF treatment into standard-of-care systemic therapy regimens. The addition of PEF to existing therapeutic regimens generates local and distant immune upregulation in this murine model, suggesting potentially improving outcomes in resectable and unresectable patient candidates, including those with immune “cold” tumors.

## Supporting information

S1 Fig Confirmation of pre-existing metastases and safe cisplatin dose. The graph indicates the recorded body weight of Naïve Balb/c mice intravenously dosed (tail vein) with 1, 2, 4, and 8 mg/kg of cisplatin once per week for 14 consecutive weeks.
(JPG)

S2 Fig Confirmation of partial ablation. A) Tumors treated with a single application of PEF were harvested 3 days post-treatment and fixed in formalin to perform histological analysis. The sample was cross-sectioned perpendicular to the needle track. H&E staining of PEF-treated tumors (B) was used to identify the cellular depletion areas in the samples. PEF-treated tumors showed partial ablation area which corresponded to 70–80% of the histology section.
(JPG)

S3 Fig Day-10 serum cytokine levels. **A)** The table reports the values of the 44 cytokines analyzed in the serum of mice that did not undergo resection and received systemic therapy alone (Cisplatin+ αPD-1) or in combination with PEF. Each group consisted of 10 mice and the p-value is reported. **B)** The graphs report the serum concentration of the tumorigenic cytokines IL-13, IL-5, and CCL5 (RANTES). Asterisks indicate the t-test p-value, *<0.05, **<0.001.
(JPG)

S4 Fig Rechallenge 4T1 tumor volumes. All the survivor mice still alive after 100 days from the PEF treatment were rechallenged in axillary fat pad with 200,000 4T1 cells. Tumors were monitored and measured three times per week until euthanasia.
(JPG)

S5 Fig Gating strategy for flow cytometry analysis. A) Gating strategy for the main peripheral blood lymphocytes. Lymphocytes were selected from a forward scatter area vs side scatter-area dot plot, and single cells were subsequently selected in a forward scatter-area vs forward scatter height dot plot. Then, T cells were selected by CD3+ expression and CD8+ cytotoxic and CD4+ helper T cells were identified by a CD8 vs CD4 dot plot. Circulating effector memory(EM) were selected from CD4+ T cells by positive staining of CD44 and negative staining of CD62L, Central memory(CM) were selected from CD4 + T cells by double positive staining of CD44 and CD62L, Naïve were selected from CD4+T cell by positive staining of CD62L and negative staining of CD44, Double negative(DN) were selected from CD4+ T cells by negative staining of CD44 and CD62L. Circulating effector memory (EM) were selected from CD8+ T cells by positive staining of CD44 and negative staining of CD62L, Central memory(CM) were selected from CD8 + T cells by double positive staining of CD44 and CD62L, Naïve were selected from CD8+T cell by positive staining of CD62L and negative staining of CD44, Double negative (DN) were selected from CD8+ T cells by negative staining of CD44 and CD62L. Activated CD4 +T cells were selected by double positive staining of CD4 and CD69. Activated CD8 +T cells were selected by double positive staining of CD8 and CD69. B) Gating strategy for GP70 Tetramer, Lymphocytes were selected from a forward scatter area vs side scatter-area dot plot, and single cells were subsequently selected in a forward scatter-area vs forward scatter height dot plot. Then, T cells were selected by CD3+ expression and CD8+ cytotoxic. Gp70 Tetramer binding to CD8 were selected with double positive staining of CD8 and gp70 tetramer.
(PDF)

S6 Fig Pulsed Electric Field (PEF) energy uses short duration, high voltage, electrical pulses to create an electric field that destabilizes cells through multiple biochemical processes. Because PEF does not rely on thermal changes to alter cells, it can be delivered to the target without damaging interstitial proteins. PEF can induce proinflammatory signaling as well as viable antigen presentation within the tumor microenvironment to promote a tumor-specific immune response that can counteract the primary treated tumor as well as distal micrometastases.
(JPG)

S1 Table Cell surface markers for characterization of immune cell types in flow cytometric analysis. (PDF)

S2 Table List of cytokines analyzed in serum. (PDF)

S3 Table Resection metastasis study tumor volume at time of resection. (PDF)

S4 Table Raw data. (PDF)
