# Supplementary figures and images for: Neoadjuvant chemo-immunotherapy is improved with a novel pulsed electric field technology in an immune-cold murine model
Source: PLoS One. 2024 Mar 25;19(3):e0299499. doi: 10.1371/journal.pone.0299499 (PMC10962799; doi:10.1371/journal.pone.0299499)

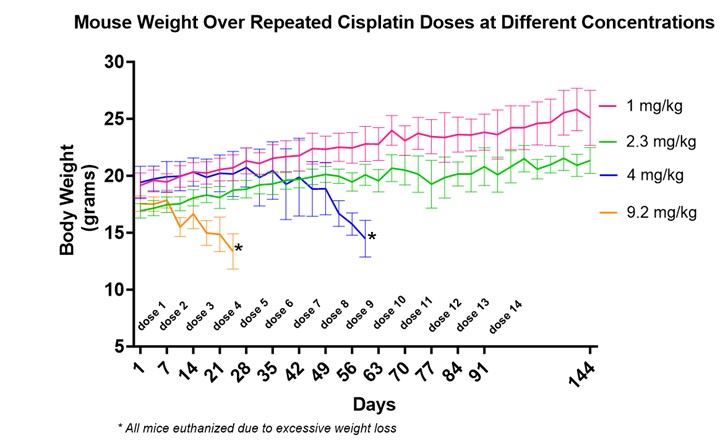

Supplement: S1 Fig — The graph indicates the recorded body weight of Naïve Balb/c mice intravenously dosed (tail vein) with 1, 2, 4, and 8 mg/kg of cisplatin once per week for 14 consecutive weeks. (JPG) [file pone.0299499.s001.jpg]

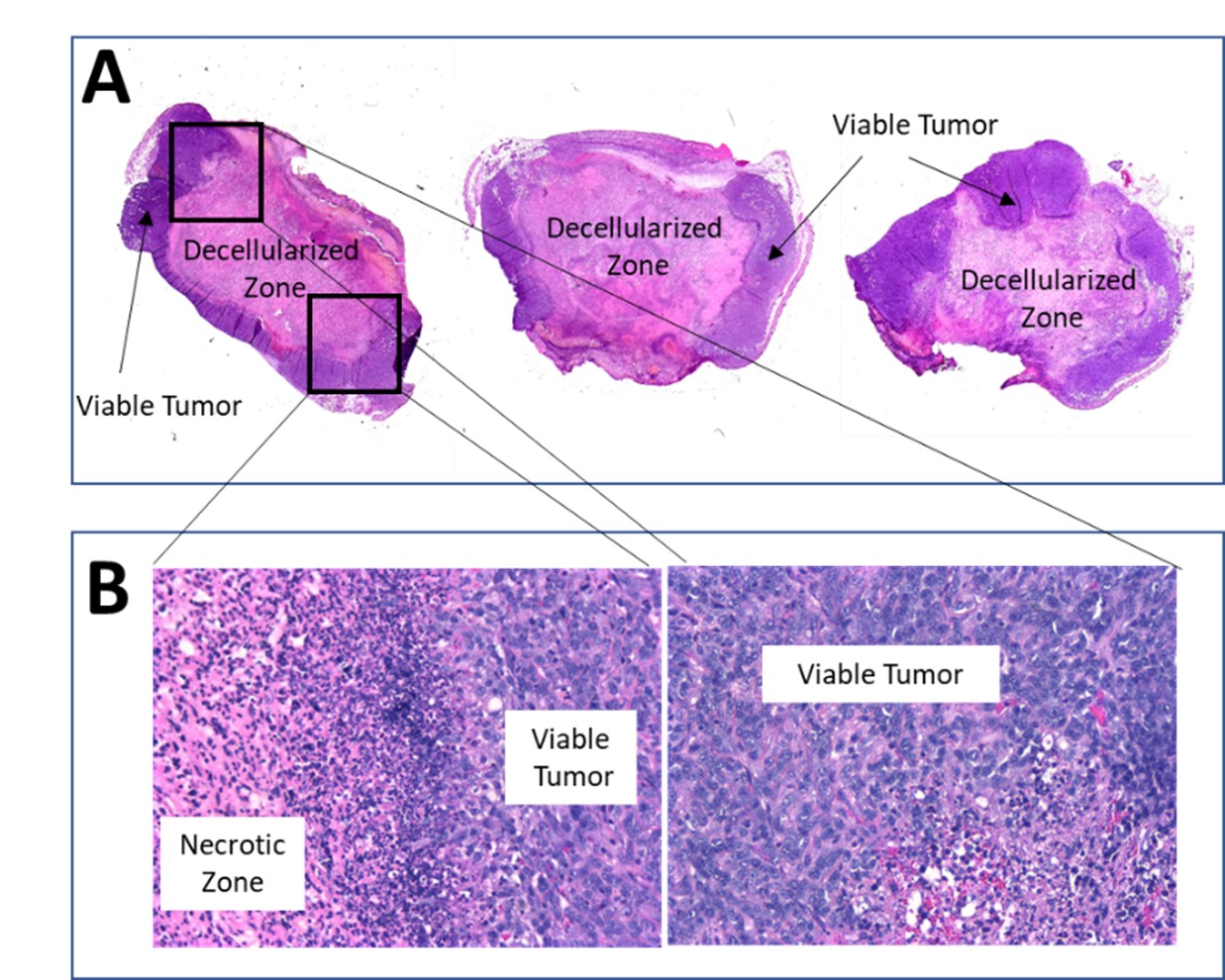

Supplement: S2 Fig — A) Tumors treated with a single application of PEF were harvested 3 days post-treatment and fixed in formalin to perform histological analysis. The sample was cross-sectioned perpendicular to the needle track. H&E staining of PEF-treated tumors (B) was used to identify the cellular depletion areas in the samples. PEF-treated tumors showed partial ablation area which corresponded to 70–80% of the histology section. (JPG) [file pone.0299499.s002.jpg]

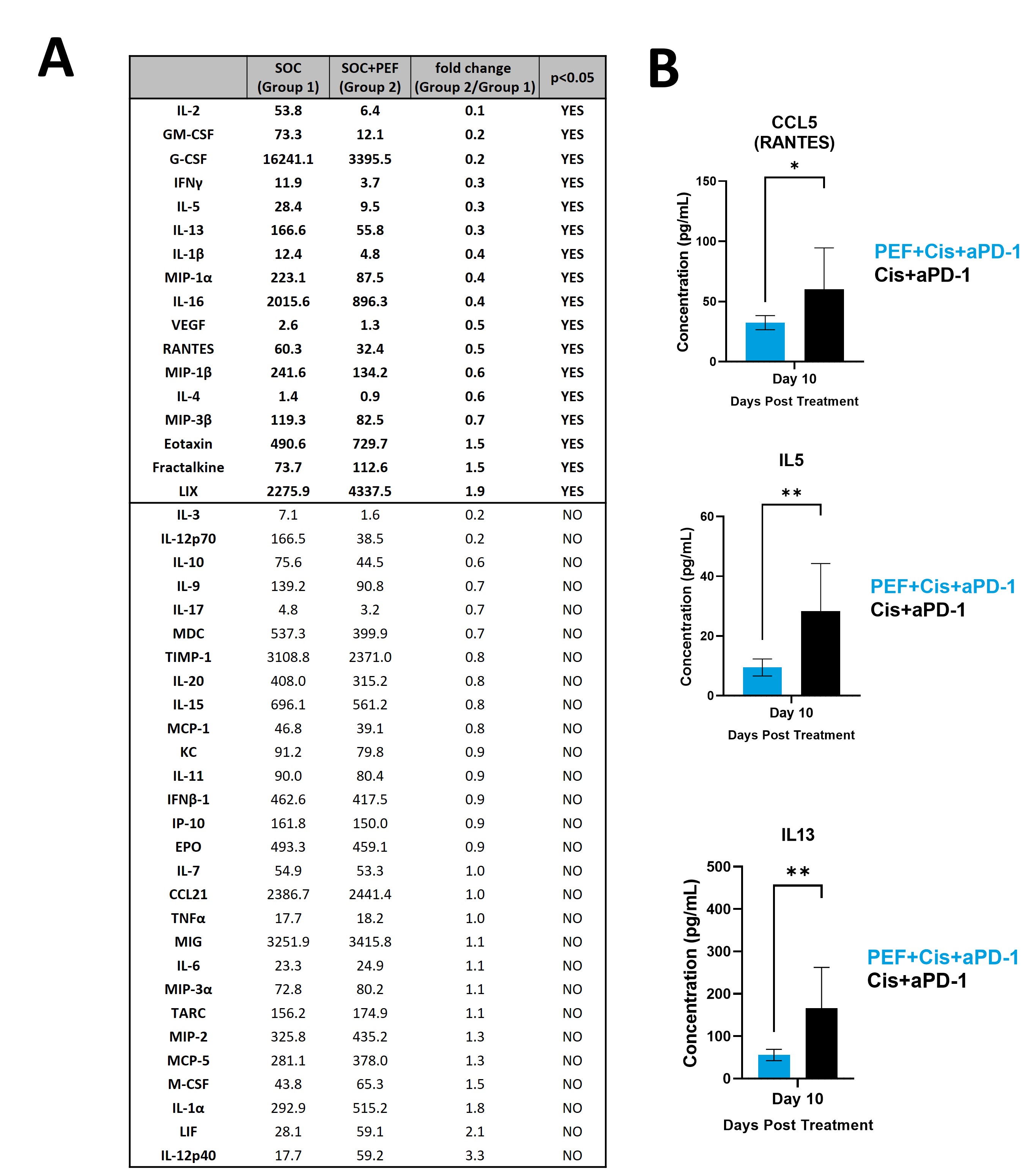

Supplement: S3 Fig — A) The table reports the values of the 44 cytokines analyzed in the serum of mice that did not undergo resection and received systemic therapy alone (Cisplatin+ αPD-1) or in combination with PEF. Each group consisted of 10 mice and the p-value is reported. B) The graphs report the serum concentration of the tumorigenic cytokines IL-13, IL-5, and CCL5 (RANTES). Asterisks indicate the t-test p-value, *<0.05, **<0.001. (JPG) [file pone.0299499.s003.jpg]

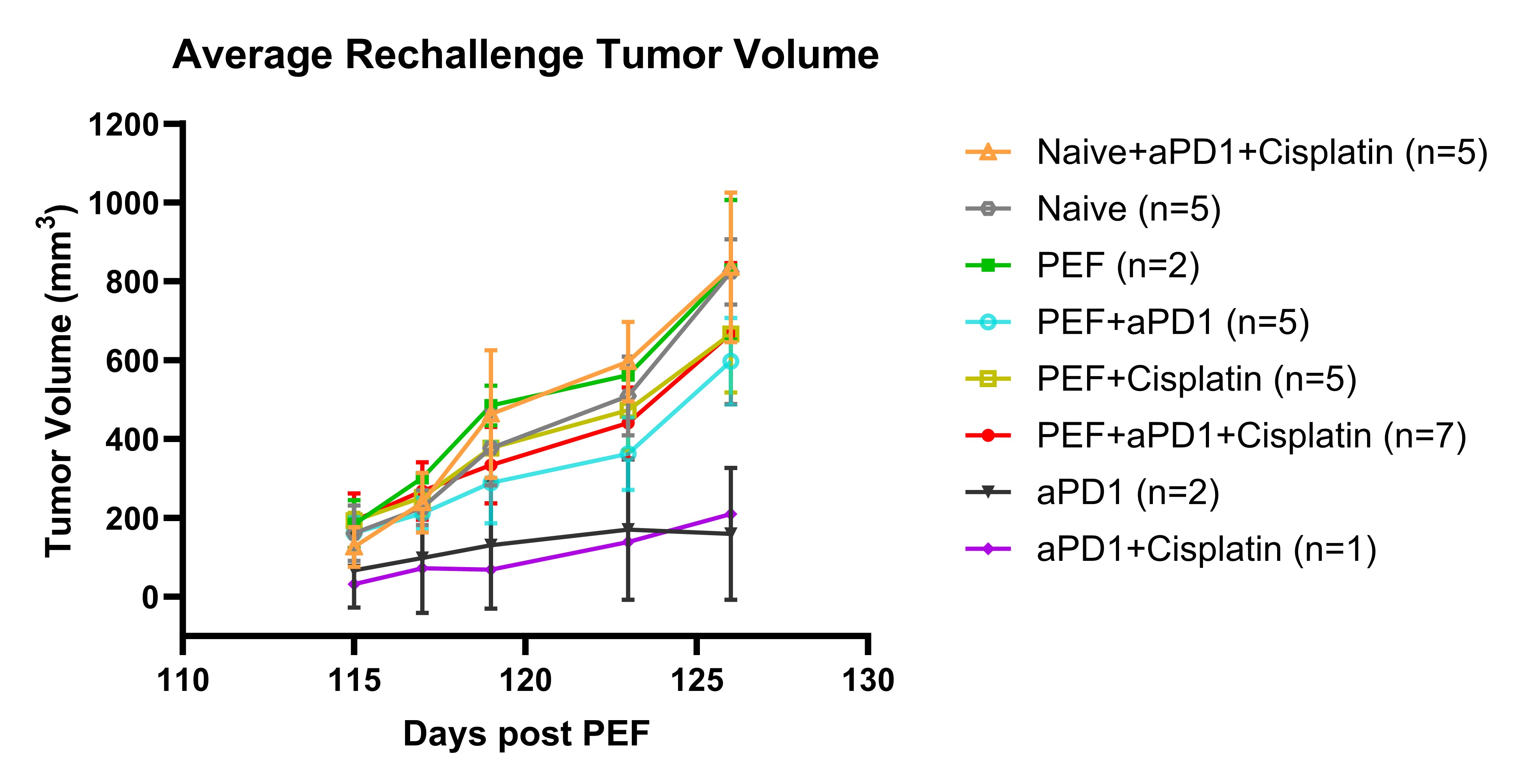

Supplement: S4 Fig — All the survivor mice still alive after 100 days from the PEF treatment were rechallenged in axillary fat pad with 200,000 4T1 cells. Tumors were monitored and measured three times per week until euthanasia. (JPG) [file pone.0299499.s004.jpg]

A

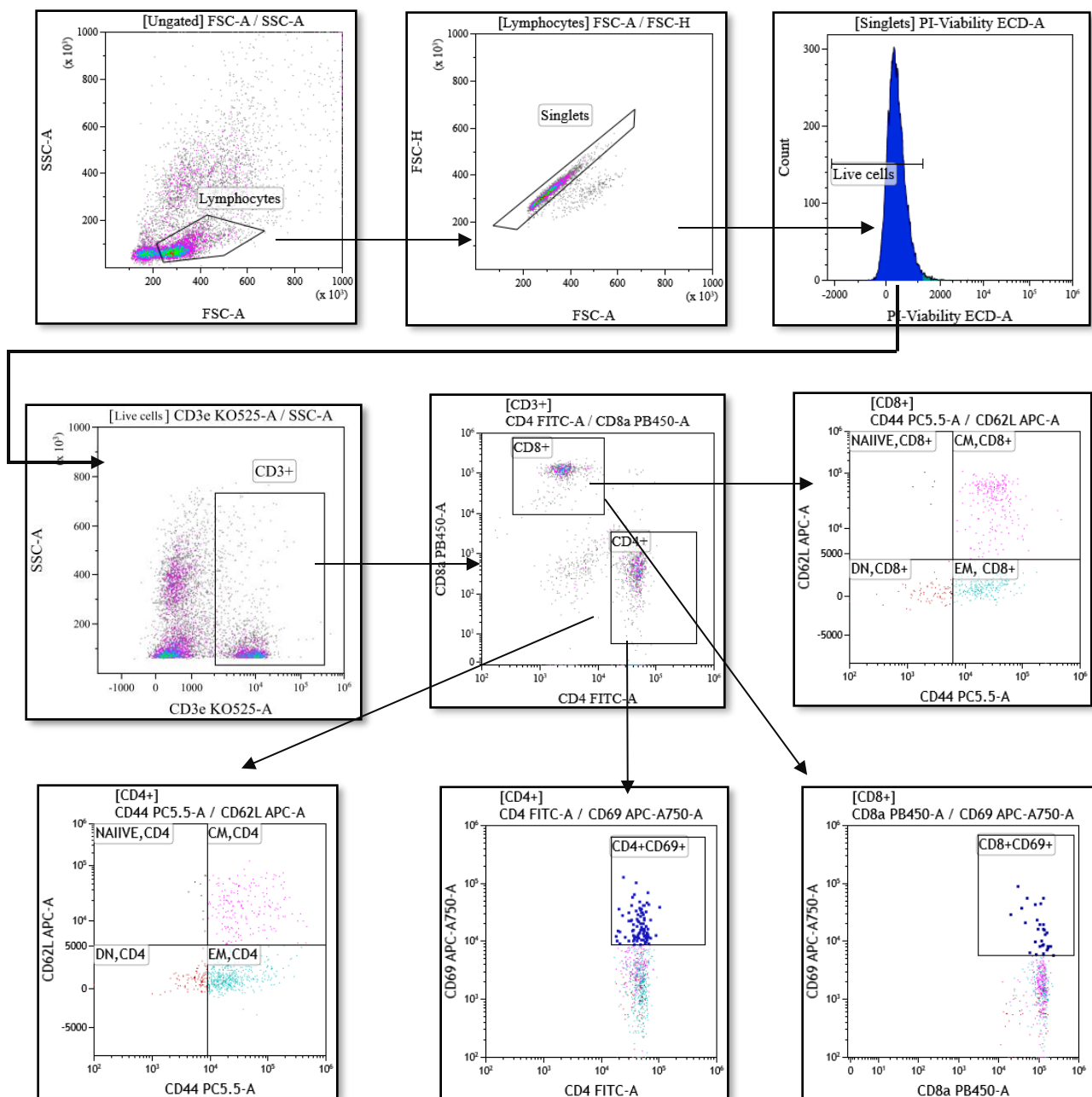

B

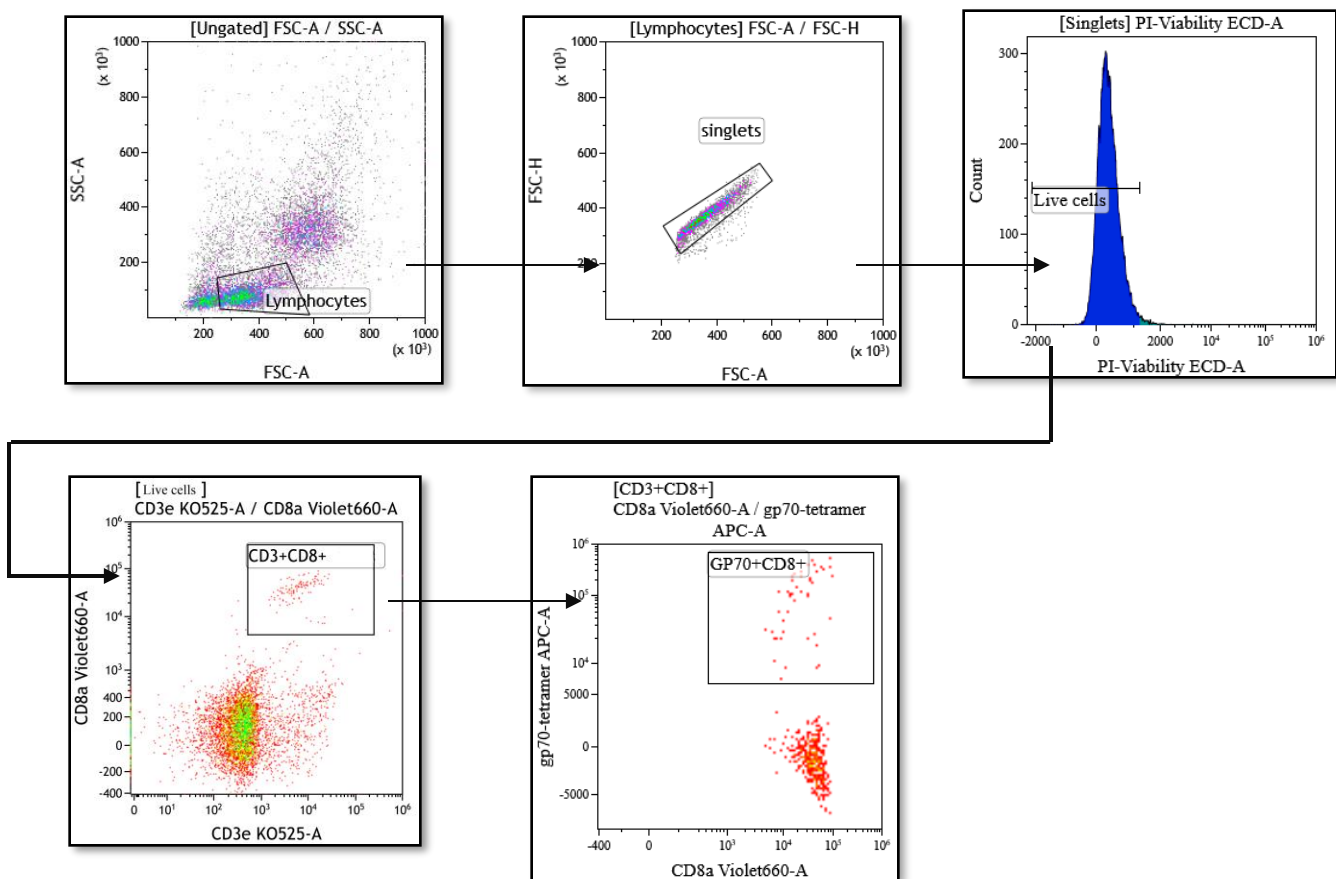

Supplement: S5 Fig — A) Gating strategy for the main peripheral blood lymphocytes. Lymphocytes were selected from a forward scatter area vs side scatter-area dot plot, and single cells were subsequently selected in a forward scatter-area vs forward scatter height dot plot. Then, T cells were selected by CD3+ expression and CD8+ cytotoxic and CD4+ helper T cells were identified by a CD8 vs CD4 dot plot. Circulating effector memory(EM) were selected from CD4+ T cells by positive staining of CD44 and negative staining of CD62L, Central memory(CM) were selected from CD4 + T cells by double positive staining of CD44 and CD62L, Naïve were selected from CD4+T cell by positive staining of CD62L and negative staining of CD44, Double negative(DN) were selected from CD4+ T cells by negative staining of CD44 and CD62L. Circulating effector memory (EM) were selected from CD8+ T cells by positive staining of CD44 and negative staining of CD62L, Central memory(CM) were selected from CD8 + T cells by double positive staining of CD44 and CD62L, Naïve were selected from CD8+T cell by positive staining of CD62L and negative staining of CD44, Double negative (DN) were selected from CD8+ T cells by negative staining of CD44 and CD62L. Activated CD4 +T cells were selected by double positive staining of CD4 and CD69. Activated CD8 +T cells were selected by double positive staining of CD8 and CD69. B) Gating strategy for GP70 Tetramer, Lymphocytes were selected from a forward scatter area vs side scatter-area dot plot, and single cells were subsequently selected in a forward scatter-area vs forward scatter height dot plot. Then, T cells were selected by CD3+ expression and CD8+ cytotoxic. Gp70 Tetramer binding to CD8 were selected with double positive staining of CD8 and gp70 tetramer. (PDF) [file pone.0299499.s005.pdf]

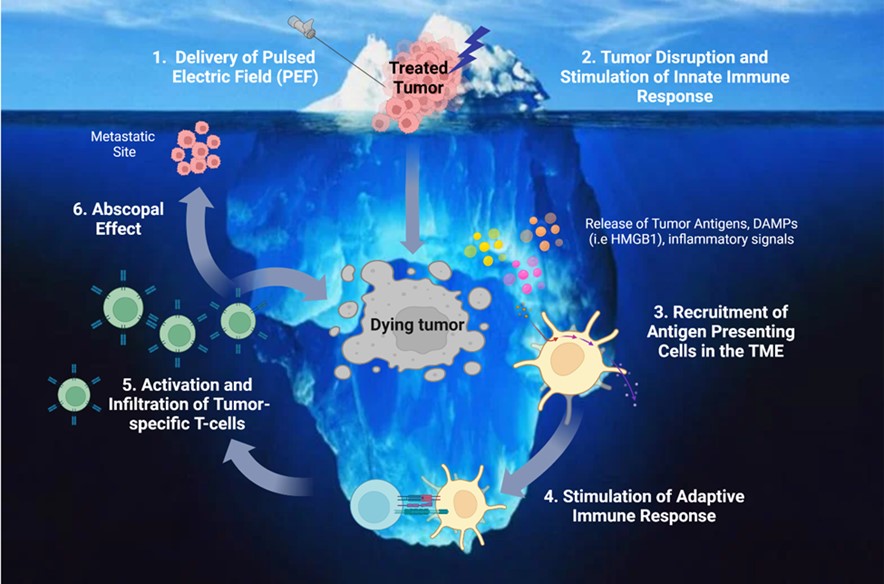

Supplement: S6 Fig — Because PEF does not rely on thermal changes to alter cells, it can be delivered to the target without damaging interstitial proteins. PEF can induce proinflammatory signaling as well as viable antigen presentation within the tumor microenvironment to promote a tumor-specific immune response that can counteract the primary treated tumor as well as distal micrometastases. (JPG) [file pone.0299499.s006.jpg]
