# Supplementary material for: Neoadjuvant chemo-immunotherapy is improved with a novel pulsed electric field technology in an immune-cold murine model
Source: PLoS One. 2024 Mar 25;19(3):e0299499. doi: 10.1371/journal.pone.0299499 (PMC10962799; doi:10.1371/journal.pone.0299499)
Supplement: S1 Table — (PDF) [file pone.0299499.s007.pdf]

Supplementary Table S1. Cell surface markers used for characterization of immune cell types in flow cytometric analysis.

**Antibody**	**Vendor and Catalog number**
Anti-mouse CD45	Biolegend, 103126
Anti-Mouse CD3ε	BD Biosciences, 563024
Anti-Mouse CD8a	Biolegend, 100738
Anti-Mouse CD4	BD Biosciences, 561828
Anti-mouse CD279 (PD-1)	Biolegend, 135225
Anti-mouse CD44	Biolegend, 103032
Anti-mouse CD69	Biolegend, 561240
Anti-Mouse CD366 (TIM-3)	Biolegend, 568905
Anti-Mouse CD25	BD Biosciences, 564021
Anti-Mouse CD62L	BD Biosciences, 553152
