# Supplementary material for: Neoadjuvant chemo-immunotherapy is improved with a novel pulsed electric field technology in an immune-cold murine model
Source: PLoS One. 2024 Mar 25;19(3):e0299499. doi: 10.1371/journal.pone.0299499 (PMC10962799; doi:10.1371/journal.pone.0299499)
Supplement: S2 Table — (PDF) [file pone.0299499.s008.pdf]

Supplementary Table S2

List of cytokines analyzed in serum
CCL11, GCSF, CSF2, IFNG, IL1A, IL1B, IL2, IL3, IL4, IL5, IL6, IL7, IL9, IL10, IL12IL13, IL15, IL17, CXCL10, CXCL1, LIF, CXCL5, CCL2, CSF1, CXCL9, CXCL3, PF4, CXCL2, CCL5, TNFA, VEGF, CCL21, EPO, CX3CL1, IFNB1, IL11, IL16, IL20, SCYA12, MDC, CCL20, CCL19, CCL17, TIMP1.
