# Supplementary material for: Neoadjuvant chemo-immunotherapy is improved with a novel pulsed electric field technology in an immune-cold murine model
Source: PLoS One. 2024 Mar 25;19(3):e0299499. doi: 10.1371/journal.pone.0299499 (PMC10962799; doi:10.1371/journal.pone.0299499)
Supplement: S3 Table — (PDF) [file pone.0299499.s009.pdf]

Supplementary Table S3. Resection Metastasis Study Tumor Volume at Time of Resection

**Experimental group**	**Mean Tumor Volume* (mm^3)**	**Statistical t-test versus Sham/IgG**
αPD-1+ cisplatin	384.7 ± 127	p=0.005
αPD-1	300.0 ± 128	p=0.14
Cisplatin	300.0 ± 36.9	p=0.16
Sham/IgG	223.3 ± 94.0	N/A
PEF	76.7 ± 25.5	p=0.0003
PEF + αPD-1	49.6 ± 15.0	p<0.0001
PEF+ cisplatin	47.1 ± 15.7	p<0.0001
PEF+ cisplatin + αPD-1	41.4 ± 20.4	p<0.0001

*Values are presented as mea
