# Supplementary material for: Neoadjuvant chemo-immunotherapy is improved with a novel pulsed electric field technology in an immune-cold murine model
Source: PLoS One. 2024 Mar 25;19(3):e0299499. doi: 10.1371/journal.pone.0299499 (PMC10962799; doi:10.1371/journal.pone.0299499)
Supplement: S4 Table — (PDF) [file pone.0299499.s010.pdf]

Figure 2A

	-2	3	5	7	10	12	14
PEF + Anti-PD1 + Cisplati	63.0568	44.84197	65.8282	86.3218	353.4503	230.7204	351.2766
PEF + Anti-PD1 + Cisplati	80.444	137.8276	26.2057	55.0041	88.72975	53.58594	61.65771
PEF + Anti-PD1 + Cisplati	90.06191	132.1719	62.78061	44.33053	75.1513	80.03854	74.00531
PEF + Anti-PD1 + Cisplati	106.1683	130.0625	76.12888	102.2092	220.5436	63.12794	357.5173
PEF + Anti-PD1 + Cisplati	90.4203	205.8314	50.07565	78.55313	47.75864	51.75625	121.0754
PEF + Anti-PD1 + Cisplati	100.5963	86.90074	61.4497	94.45414	203.6325	300.8872	413.7174
PEF + Anti-PD1 + Cisplati	57.05344	48.42577	37.49402	52.83245	68.92844	41.90839	57.97154
PEF + Anti-PD1 + Cisplati	63.63176	115.0245	31.15477	40.83248	49.8318	44.23972	77.08187
PEF + Anti-PD1 + Cisplati	74.79778	90.19638	37.2438	48.7842	46.1727	28.54858	74.01795
PEF + Anti-PD1 + Cisplati	127.2306	59.92103	22.35989	63.30097	53.92509	22.74965	61.01197
Anti-PD1 + Cisplatin	52.76891	188.4939	259.3366	180.2922	285.0734	238.1438	355.4291
Anti-PD1 + Cisplatin	117.8093	293.9328	395.5285	591.8352	534.4626	741.0463	913.0711
Anti-PD1 + Cisplatin	96.29205	162.0171	372.7686	365.3645	523.4708	854.3693	1041.951
Anti-PD1 + Cisplatin	91.25885	193.3002	460.5822	373.5745	589.6554	413.1027	699.7685
Anti-PD1 + Cisplatin	62.28093	169.6823	566.1722	735.9288	846.4793	593.756	931.8699
Anti-PD1 + Cisplatin	58.48366	147.6013	280.4613	427.0073	624.7632	637.1966	364.3652
Anti-PD1 + Cisplatin	110.9738	267.6715	509.6235	858.317	751.6514	510.9206	971.3561
Anti-PD1 + Cisplatin	104.8081	243.8934	274.929	418.2422	714.8356	696.7982	927.5679
Anti-PD1 + Cisplatin	87.17684	271.2785	321.5612	353.9333	467.6578	514.4349	660.5012
Anti-PD1 + Cisplatin	86.27063	374.4891	491.2176	479.6394	545.3881	518.0751	790.4146

Figure 2B

	PEF + Anti-PD1 + Cisplatin									
Days Post T	1	2	3	4	5	6	7	8	9	10
-2	63.0568	Excluded	90.06191	106.1683	90.4203	100.5963	57.05344	63.63176	74.79778	127.2306
3	44.84197	Excluded	132.1719	130.0625	205.8314	86.90074	48.42577	115.0245	90.19638	59.92103
5	65.8282	Excluded	62.78061	76.12888	50.07565	61.4497	37.49402	31.15477	37.2438	22.35989
7	86.3218	Excluded	44.33053	102.2092	78.55313	94.45414	52.83245	40.83248	48.7842	63.30097
10	353.4503	Excluded	75.1513	220.5436	47.75864	203.6325	68.92844	49.8318	46.1727	53.92509
12	230.7204	Excluded	80.03854	63.12794	51.75625	300.8872	41.90839	44.23972	28.54858	22.74965
14	351.2766	Excluded	74.00531	357.5173	121.0754	413.7174	57.97154	77.08187	74.01795	61.01197
17	323.0405	Excluded	138.7913	947.7175	198.3106	523.6279	21.2126	0	38.9215	21.1548
19	482.4924	Excluded	264.8545	894.5288	280.5905	753.8216	0	0	0	0
21	289.5944	Excluded	286.8264	1029.601	388.9027	914.4209	42.525	0	0	0
24	1475.435	Excluded	287.0751	1380.905	601.1363		0	0	0	0
26	1521.113	Excluded	484.1913	1647.603	649.8231		0	0	0	0
31	1934	Excluded	533		864		0	0	0	0
33		Excluded	617		987		0	0	0	136
38		Excluded	1703		1596		0	0	0	247
40		Excluded					0	0	0	341.7
42		Excluded					0	0	0	475.5
45		Excluded					152	0	0	987.4
47		Excluded					260.3	0	0	1025.3
49		Excluded					407	0	0	1376.9
52		Excluded					508.1	0	0	1545.4

	SOC									
Days Post T	1	2	3	4	5	6	7	8	9	10
-2	52.76891	117.8093	96.29205	91.25885	62.28093	58.48366	110.9738	104.8081	87.17684	86.27063
3	188.4939	293.9328	162.0171	193.3002	169.6823	147.6013	267.6715	243.8934	271.2785	374.4891
5	259.3366	395.5285	372.7686	460.5822	566.1722	280.4613	509.6235	274.929	321.5612	491.2176
7	180.2922	591.8352	365.3645	373.5745	735.9288	427.0073	858.317	418.2422	353.9333	479.6394
10	285.0734	534.4626	523.4708	589.6554	846.4793	624.7632	751.6514	714.8356	467.6578	545.3881
12	238.1438	741.0463	854.3693	413.1027	593.756	637.1966	510.9206	696.7982	514.4349	518.0751
14	355.4291	913.0711	1041.951	699.7685	931.8699	364.3652	971.3561	927.5679	660.5012	790.4146
17	528.1656			1451.782		777.8739		840.367	1164.87	1116.847
19	806.8703			1786.061		1094.928		1304.797	1093.546	1127.819
21	799.8428			1465.987		1562.458		1628.111	1589.108	1565.416
24										
26										
31										
33										
38										
40										
42										
45										
47										
49										
52										

Figure 2C No-Resection Cohorts

mouse/cage	Day Euthanized	PEF + Anti-PD1 + Cisplatin	Anti-PD1 + Cisplatin
9(1)	31	1	
9(3)	38	1	
9(4)	28	1	
9(5)	38	1	
10(1)	24	1	
10(2)	52	0	
10(3)	52	0	
10(4)	52	0	
10(5)	52	0	
19(1)	24		1
19(2)	14		1
19(3)	14		1
19(4)	24		1
19(5)	14		1
20(1)	24		1
20(2)	17		1
20(3)	24		1
20(4)	24		1
20(5)	24		1

Figure 3- T cells

	CD3+	CD8+	CD4+
PEF + Anti-PD1 + Cisplatin -- No Resection	12.22	3.66	7.87
PEF + Anti-PD1 + Cisplatin -- No Resection	30.1	7.96	19.47
PEF + Anti-PD1 + Cisplatin -- No Resection	18.55	5.32	12.37
PEF + Anti-PD1 + Cisplatin -- No Resection	12.77	3.44	8.46
PEF + Anti-PD1 + Cisplatin -- No Resection	19.45	6.1	10.36
PEF + Anti-PD1 + Cisplatin -- No Resection	13.02	3.97	8.18
PEF + Anti-PD1 + Cisplatin -- No Resection	29.89	8.13	20.46
PEF + Anti-PD1 + Cisplatin -- No Resection	25.95	6.9	17.33
PEF + Anti-PD1 + Cisplatin -- No Resection	26.57	6.88	17.74
PEF + Anti-PD1 + Cisplatin -- No Resection	34.54	8	24.12
Anti-PD1 + Cisplatin -- No Resection	6.87	1.81	4.56
Anti-PD1 + Cisplatin -- No Resection	4.39	1.19	2.88
Anti-PD1 + Cisplatin -- No Resection	5.04	1.67	3.19
Anti-PD1 + Cisplatin -- No Resection	7.37	1.9	4.8
Anti-PD1 + Cisplatin -- No Resection	6.61	1.74	4.12
Anti-PD1 + Cisplatin -- No Resection	11.64	3.28	7.73
Anti-PD1 + Cisplatin -- No Resection	7.63	2.16	4.49
Anti-PD1 + Cisplatin -- No Resection	5.77	1.57	3.13
Anti-PD1 + Cisplatin -- No Resection	8.42	1.95	5.85
Anti-PD1 + Cisplatin -- No Resection	4.37	1.06	2.82

Figure 3- CD8 subsets

	Naïve	CM	EM	DN
PEF + Anti-PD1 + Cisplatin -- No Resection	0.63	18.73	49.21	31.43
PEF + Anti-PD1 + Cisplatin -- No Resection	1.23	16.8	55.47	26.5
PEF + Anti-PD1 + Cisplatin -- No Resection	0.25	12.99	65.93	20.83
PEF + Anti-PD1 + Cisplatin -- No Resection	1.6	17.6	48.8	32
PEF + Anti-PD1 + Cisplatin -- No Resection	0	13.88	66.6	19.51
PEF + Anti-PD1 + Cisplatin -- No Resection	4.01	35.19	32.72	28.09
PEF + Anti-PD1 + Cisplatin -- No Resection	0.77	7.36	53.68	38.19
PEF + Anti-PD1 + Cisplatin -- No Resection	1.65	15.36	49.18	33.82
PEF + Anti-PD1 + Cisplatin -- No Resection	1.07	10.18	50.36	38.39
PEF + Anti-PD1 + Cisplatin -- No Resection	0.9	8.3	56.26	34.54
Anti-PD1 + Cisplatin -- No Resection	1.25	1.25	46.25	51.25
Anti-PD1 + Cisplatin -- No Resection	0	0.91	47.27	51.82
Anti-PD1 + Cisplatin -- No Resection	0	1.31	56.86	41.83
Anti-PD1 + Cisplatin -- No Resection	0	0	43.6	56.4
Anti-PD1 + Cisplatin -- No Resection	0	0.63	49.37	50
Anti-PD1 + Cisplatin -- No Resection	4.08	3.4	44.22	48.3
Anti-PD1 + Cisplatin -- No Resection	0.52	3.63	59.07	36.79
Anti-PD1 + Cisplatin -- No Resection	0	1.4	62.94	35.66
Anti-PD1 + Cisplatin -- No Resection	0.59	0	43.2	56.21
Anti-PD1 + Cisplatin -- No Resection	0	0	37.89	62.11

Figure 3- CD4 subsets

	Naïve	CM	EM	DN
PEF + Anti-PD1 + Cisplatin -- No Resection	1.33	9.01	73.12	16.54
PEF + Anti-PD1 + Cisplatin -- No Resection	1.07	6.61	77.46	14.86
PEF + Anti-PD1 + Cisplatin -- No Resection	0.32	7.81	79.64	12.24
PEF + Anti-PD1 + Cisplatin -- No Resection	0.81	11.06	74.96	13.17
PEF + Anti-PD1 + Cisplatin -- No Resection	1.33	9.83	78.01	10.83
PEF + Anti-PD1 + Cisplatin -- No Resection	3.14	19.61	63.62	13.62
PEF + Anti-PD1 + Cisplatin -- No Resection	0.98	6.7	78.92	13.41
PEF + Anti-PD1 + Cisplatin -- No Resection	1.53	6.99	79.24	12.24
PEF + Anti-PD1 + Cisplatin -- No Resection	0.55	6.79	78.25	14.4
PEF + Anti-PD1 + Cisplatin -- No Resection	1.25	6.45	78.2	14.1
Anti-PD1 + Cisplatin -- No Resection	0.5	0.74	49.63	49.13
Anti-PD1 + Cisplatin -- No Resection	0.38	1.13	59.62	38.87
Anti-PD1 + Cisplatin -- No Resection	0	1.71	86.35	11.95
Anti-PD1 + Cisplatin -- No Resection	0	0.92	60.14	38.94
Anti-PD1 + Cisplatin -- No Resection	0	1.07	55.2	43.73
Anti-PD1 + Cisplatin -- No Resection	0.29	1.01	63.2	35.5
Anti-PD1 + Cisplatin -- No Resection	0	1.25	73.82	24.94
Anti-PD1 + Cisplatin -- No Resection	0	0.7	79.37	19.93
Anti-PD1 + Cisplatin -- No Resection	0.2	1.19	55.14	43.48
Anti-PD1 + Cisplatin -- No Resection	0.4	1.19	55.95	42.46

Figure 3- activated T cells

	CD4+	CD8+
PEF + Anti-PD1 + Cisplatin -- No Resection	11376.5	24741.3
PEF + Anti-PD1 + Cisplatin -- No Resection	8760.1	29892.4
PEF + Anti-PD1 + Cisplatin -- No Resection	9504.1	23044.8
PEF + Anti-PD1 + Cisplatin -- No Resection	8546.1	21142
PEF + Anti-PD1 + Cisplatin -- No Resection	8467	20541.2
PEF + Anti-PD1 + Cisplatin -- No Resection	8096.1	25563.3
PEF + Anti-PD1 + Cisplatin -- No Resection	12480.5	28286.4
PEF + Anti-PD1 + Cisplatin -- No Resection	8211	25044.6
PEF + Anti-PD1 + Cisplatin -- No Resection	8833.8	22823.7
PEF + Anti-PD1 + Cisplatin -- No Resection	11130.3	30042.1
Anti-PD1 + Cisplatin -- No Resection	7689.9	19054.3
Anti-PD1 + Cisplatin -- No Resection	6186.9	15073.9
Anti-PD1 + Cisplatin -- No Resection	7457.2	13516.2
Anti-PD1 + Cisplatin -- No Resection	5138.2	13221.1
Anti-PD1 + Cisplatin -- No Resection	4894.3	13043.9
Anti-PD1 + Cisplatin -- No Resection	6532.3	14389.6
Anti-PD1 + Cisplatin -- No Resection	7060.3	16582.2
Anti-PD1 + Cisplatin -- No Resection	7654.9	17978.3
Anti-PD1 + Cisplatin -- No Resection	6036.6	13643.3
Anti-PD1 + Cisplatin -- No Resection	5694.9	16776.8

Figure 3- antigen specific T cells

	MFI
PEF + Anti-PD1 + Cisplatin -- No Resection	11179.9
PEF + Anti-PD1 + Cisplatin -- No Resection	3712.9
PEF + Anti-PD1 + Cisplatin -- No Resection	3457.3
PEF + Anti-PD1 + Cisplatin -- No Resection	5052.7
PEF + Anti-PD1 + Cisplatin -- No Resection	4327
PEF + Anti-PD1 + Cisplatin -- No Resection	3395.1
PEF + Anti-PD1 + Cisplatin -- No Resection	2702.2
PEF + Anti-PD1 + Cisplatin -- No Resection	5376.2
PEF + Anti-PD1 + Cisplatin -- No Resection	3362.8
PEF + Anti-PD1 + Cisplatin -- No Resection	9616.7
Anti-PD1 + Cisplatin -- No Resection	11988
Anti-PD1 + Cisplatin -- No Resection	13142.5
Anti-PD1 + Cisplatin -- No Resection	7569.3
Anti-PD1 + Cisplatin -- No Resection	16362.6
Anti-PD1 + Cisplatin -- No Resection	7554.6
Anti-PD1 + Cisplatin -- No Resection	8319.1
Anti-PD1 + Cisplatin -- No Resection	8948.4
Anti-PD1 + Cisplatin -- No Resection	14005.5
Anti-PD1 + Cisplatin -- No Resection	13399.8
Anti-PD1 + Cisplatin -- No Resection	11008.5

Figure 3B Antigen-specific T-cells and Tumor Volume Correlation

Survival	PEF+ Cisplatin+Anti-PD1	Anti-PD1 + Cisplatin
323.0405	3.66	
75	7.96	
138.7913	5.32	
947.7175	3.44	
198.3106	6.1	
523.6279	3.97	
21.2126	8.13	
0	6.9	
38.9215	6.88	
21.1548	8	
355.42912		1.81
913.07108		1.19
1041.9506		1.67
699.76848		1.9
931.86985		1.74
364.36518		3.28
971.35605		2.16
927.56794		1.57
660.50122		1.95
790.41461		1.06

Figure 4A - Tumor Volume Before Resection

Days Post-Treatment	-2	3	5
PEF	83.34976	182.9269	76.68547
PEF + Anti-PD1	86.33577	224.204	49.55421
PEF + Cisplatin	94.57414	123.8616	47.13391
PEF + Anti-PD1 + Cisplatin	91.75406	115.3866	41.43161
Sham/ IgG	90.34547	134.1652	223.2994
Cisplatin	89.11329	166.7045	302.9418
Anti-PD1	90.44679	197.3001	300.0893
Anti-PD1 + Cisplatin	86.93857	257.0778	384.7739

Figure 4B - Resection Mice Survival Analysis

Mouse	Time (Day)	PEF	PEF + Anti-	PEF + Cispl	PEF + Anti-	Sham/ IgG	Cisplatin	Anti-PD1	Anti-PD1 + Cisplatin
1	45	1							
2	3	1							
3	49	1							
4	52	1							
5	100	0							
6	52	1							
7	32	1							
8	63	1							
9	45	1							
10	100	0							
1	27		1						
2	100		0						
3	100		0						
4	100		0						
5	100		0						
6	77		1						
7	Excluded		0						
8	40		1						
9	100		0						
10	31		1						
1	100			0					
2	100			0					
3	100			0					
4	77			1					
5	77			1					
6	63			1					
7	100			0					
8	100			0					
9	Excluded			0					
10	40			1					
1	100				0				
2	100				0				
3	100				0				
4	100				0				
5	100				0				
6	40				1				
7	100				0				
8	31				1				
9	100				0				
10	100				0				
1	33					1			
2	40					1			
3	38					1			
4	33					1			
5	38					1			
6	38					1			
7	27					1			

8	38	1			
9	33	1			
10	27	1			
1	Excluded		1		
2	42		1		
3	38		1		
4	40		1		
5	40		1		
6	31		1		
7	Excluded		1		
8	38		1		
9	38		1		
10	Excluded		0		
1	38			1	
2	31			1	
3	52			1	
4	32			1	
5	67			1	
6	100			0	
7	100			0	
8	39			1	
9	49			1	
10	31			1	
1	49				1
2	32				1
3	39				1
4	67				1
5	49				1
6	59				1
7	63				1
8	49				1
9	67				1
10	100				0

Figure 4D - Rechallenge Tumor Volumes

PEF+Cis+αPD1 Survivors	0.140722
PEF+Cis+αPD1 Survivors	0.073387
PEF+Cis+αPD1 Survivors	0.137316
PEF+Cis+αPD1 Survivors	0.304937
PEF+Cis+αPD1 Survivors	1.066227
PEF+Cis+αPD1 Survivors	1.71532
PEF+Cis+αPD1 Survivors	0.026155
Control (2, SOC): Naive inoculation, Cis+αPD1	0.528219
Control (2, SOC): Naive inoculation, Cis+αPD1	2.056345
Control (2, SOC): Naive inoculation, Cis+αPD1	1.132695
Control (2, SOC): Naive inoculation, Cis+αPD1	1.180085
Control (2, SOC): Naive inoculation, Cis+αPD1	1.068949
Control (1):Naive inoculation, untreated	2.329605
Control (1):Naive inoculation, untreated	3.165376
Control (1):Naive inoculation, untreated	1.11313
Control (1):Naive inoculation, untreated	4.63866
Control (1):Naive inoculation, untreated	1.536379
